# Reconstructing aspects of human embryogenesis with pluripotent stem cells

**DOI:** 10.1038/s41467-021-25853-4

**Published:** 2021-09-21

**Authors:** Berna Sozen, Victoria Jorgensen, Bailey A. T. Weatherbee, Sisi Chen, Meng Zhu, Magdalena Zernicka-Goetz

**Affiliations:** 1 Plasticity and Self-Organization Group, Division of Biology and Biological Engineering, Caltech, Pasadena, CA 91125 USA; 2 Department of Genetics, Yale School of Medicine, Yale University, New Haven, CT 06520 USA; 3 Mammalian Development and Stem Cell Group, Department of Physiology, Development and Neuroscience, University of Cambridge, Cambridge, CB2 3EG UK; 4 Blavatnik Institute, Harvard Medical School, Department of Genetics, Boston, MA 02115 USA

**Keywords:** Cell biology, Cell polarity, Embryogenesis, Morphogenesis, Totipotent stem cells

## Abstract

Understanding human development is of fundamental biological and clinical importance. Despite its significance, mechanisms behind human embryogenesis remain largely unknown. Here, we attempt to model human early embryo development with expanded pluripotent stem cells (EPSCs) in 3-dimensions. We define a protocol that allows us to generate self-organizing cystic structures from human EPSCs that display some hallmarks of human early embryogenesis. These structures mimic polarization and cavitation characteristic of pre-implantation development leading to blastocyst morphology formation and the transition to post-implantation-like organization upon extended culture. Single-cell RNA sequencing of these structures reveals subsets of cells bearing some resemblance to epiblast, hypoblast and trophectoderm lineages. Nevertheless, significant divergences from natural blastocysts persist in some key markers, and signalling pathways point towards ways in which morphology and transcriptional-level cell identities may diverge in stem cell models of the embryo. Thus, this stem cell platform provides insights into the design of stem cell models of embryogenesis.

## Abstract (web summary)

Human early development remains largely inaccessible, owing to technical and ethical limitations of working with natural embryos. Here the authors assess the extent to which human expanded pluripotent stem cells can specify distinct cell lineages and capture aspects of early human embryogenesis.

## Introduction

**Fig. 1:**
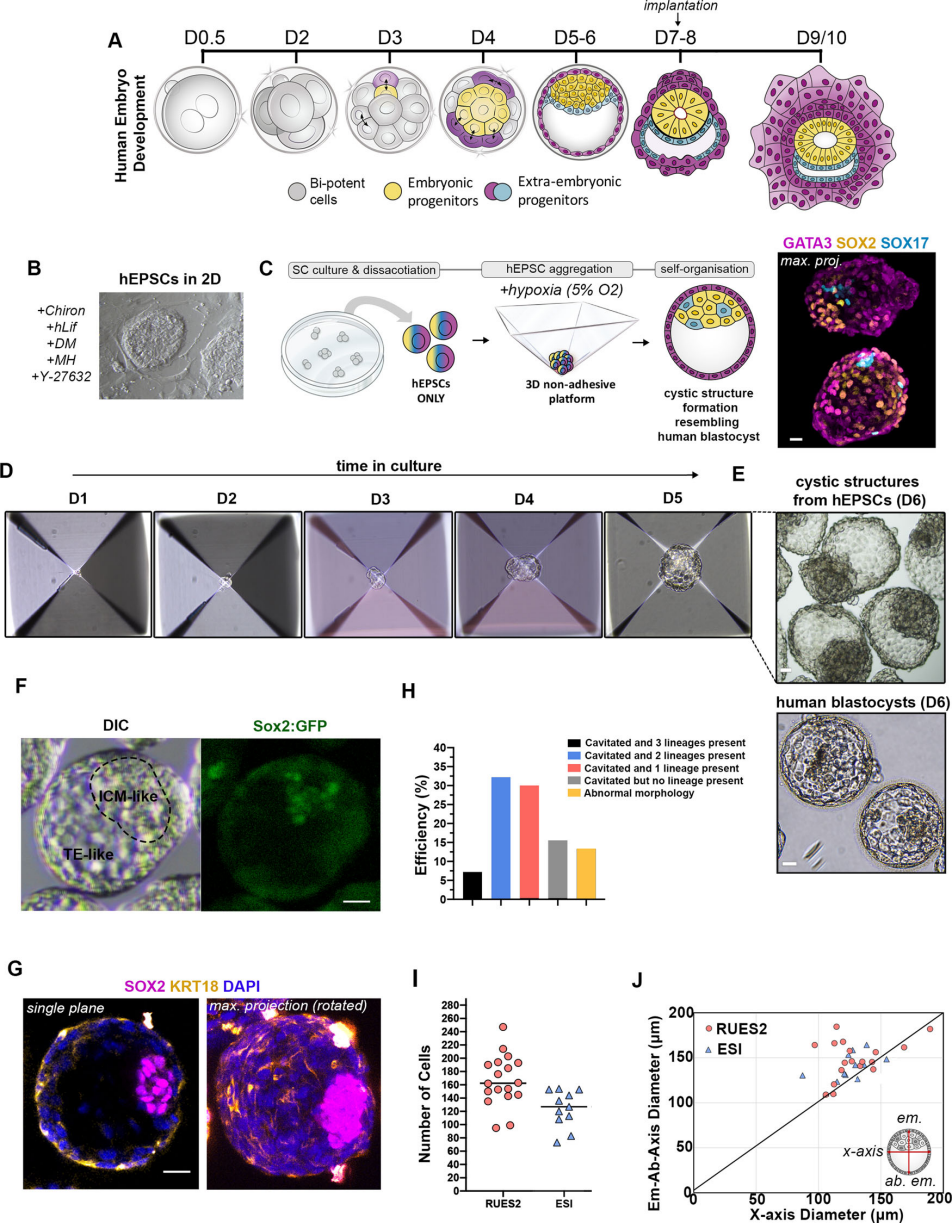
A 3D system from hEPSCs to mimic embryo-like events. **A** Scheme for natural human pre/peri-implantation embryo development. **B** A representative dome-shaped naïve pluripotent hEPSC colony in 2D culture. Representative of at least 10 independent experiments. **C** Left: Schematic of an AggreWell and 3D aggregation protocol with hEPSCs. Right: Representative examples of cystic structures generated from a typical experiment after 4 days demonstrate three lineages, resembling blastocyst stage natural embryo. Representative of at least 3 independent experiments. Lineage markers: SOX2, yellow; GATA3, magenta, and SOX17, cyan. *n* = 10 experiments. **D** Representative phase-contrast images of hEPSC multicellular aggregates in AggreWell at the indicated time points during 3D culture. **E** Phase-contrast images of cystic structures from hEPSCs at D6. (top) and natural human blastocysts at D6 (bottom). Representative of at least 3 independent experiments. **F** A representative cystic structure generated from RUES2 hEPSC line with SOX2-flourescent reporter. Representative of at least 2 independent experiments. **G** A representative structure immunostained for SOX2 in magenta, KRT18 in yellow to label inner compartment and outside epithelium, respectively. DAPI is shown in blue. Maximum projection image is shown on the right. *n* = 50 structures, 3 experiments. **H** Efficiency quantification showing the number of structures with a cavity and identifiable lineage segregation. Present EPI-like and HYPO-like cells were determined by positive expression of SOX2 and SOX17, respectively, within inner compartment as seen by IF staining. Present TE-like cells were determined by positive expression of GATA3 or KRT18 in outer cells observed by IF staining. *n* = 186 structures, 2 experiments. **I** Quantification for cell numbers in individual cystic structures generated from two established hEPSC lines, ESI017 (*n* = 11), RUES2 (*n* = 18). **J** Measurements of axial diameters in cystic structures from ESI017 (*n* = 11), RUES2 (*n* = 18) hEPSC lines. Illustration on right shows the two axes measured. All scale bars in the figure indicate 20 µm.

Human life starts at fertilisation with the union of the sperm and the egg to form the zygote. This unique totipotent cell undergoes continuous cleavage divisions without any increase in size, resulting in a sphere known as the morula four days after fertilisation. Further division and cell differentiation results in a hollowed structure known as the blastocyst on the fifth day of development (Fig. 1A). By the blastocyst stage, two main groups of cells become defined: the inner cell mass (ICM), which will form the embryo proper and the first extra-embryonic tissue, the trophectoderm (TE), an epithelium that gives rise to the cells of the placenta. Just before embryo implantation on the sixth day, the inner cell mass starts to differentiate into the epiblast (EPI) and hypoblast (HYPO), which will give rise to all embryonic cells and the extra-embryonic yolk-sac, respectively^1,2^. Following implantation, the EPI undergoes a series of morphological changes leading to the formation of a 3-dimensional (3D) rosette, which then forms a flattened disc-shaped structure that will initiate gastrulation on day fourteen.

Naturally, the development of the human embryo occurs within the body of the mother, making it hard to study. Although recent in vitro culture methods have advanced our abilities to study aspects of human embryo development ex-utero^3–5^, surplus human embryos donated to research are rare and their use is subject to considerable ethical and legal restrictions^6^. Due to these reasons, knowledge of the critical developmental steps allowing formation of the blastocyst stage embryo and its subsequent remodelling at early post-implantation remain largely unknown. Thus, the generation of several stem cell-derived models that recapitulate unique stages of mouse^7–16^ and human embryo^17–23^ development in vitro have been invaluable.

Here, with these considerations in mind, we test the hypothesis that human pluripotent stem cells (hPSCs) under certain conditions could undergo self-organisation into 3D embryo-like structures. Recent studies showed that PSCs can be reprogrammed to a molecular state, termed extended or expanded pluripotency (EP), that has developmental potency for both embryonic and extra-embryonic cell lineages^24–27^. We have therefore asked whether hPSCs that are grown under EP conditions (termed hEPSCs) and cultured with a combination of appropriate growth factors and/or inhibitors can capture aspects of early embryonic lineage development in 3D culture. We show that the resulting structures recapitulate some of the morphological and gene expression features of embryonic days 3 to day 9/10 of natural human embryogenesis with limited developmental potential. Single-cell RNA sequencing (scRNA-seq) further confirms that these structures recapitulate some aspects of blastocyst gene expression, with notable divergences. We anticipate that the future applications of this system can give insight into regulatory processes of cellular differentiation in human embryo development whilst also highlighting ongoing challenges both in specifically understanding the multi-potency state of EPSCs and broadly modelling human embryogenesis in vitro.

## Results

### Self-organization of human EPSCs

We first converted hPSCs to hEPSCs through a minimum of 5 passages (Materials and Methods). The resulting cells acquired some morphological features characteristic of pluripotent cells in the naïve state of pluripotency, including dome-shaped colony formation, as supported by earlier observations^25^ (Fig. 1B). However, we could also observe flat cell colonies, a morphological feature characteristic of pluripotent cells in the primed state, present in different ratios after each passage, suggesting the presence of a mixed population of cells in different pluripotent states under EP culture conditions (Figure S1A). Using a multi-inverted-pyramidal microwell-based 3D culture system that we previously described to facilitate self-organisation of mouse embryonic and extra-embryonic stem cells^11,13,28^, we seeded small numbers of hEPSCs (5-6 cells per microwell) to enable their aggregation and subsequent self-organisation (Fig. 1C). We first observed that the in vitro culture media normally used for the culture of natural human pre-implantation embryos promoted the formation of cavitated cystic structures (Figure S1B, see Methods). Aiming to support the maintenance of pluripotency and to promote TE-like differentiation, we mixed 2 parts of this medium with 1 part of EP^25^ and 1 part of hTSC^29^, two different stem cell base media (without the addition of any growth factors or inhibitors, see Materials and Methods). We observed that conditions of low oxygen tension (5% O_2_, similar to our previous conditions for mouse blastoid formation^11^ and for the development of natural human blastocysts^30^) facilitated the formation of cavitated structures (Figure S1C).

We next screened various growth factors, cytokines, and small molecules at widely adopted concentrations^19,21^ as previously published, and to identify conditions facilitating cavity and early lineage formation in these structures (Figure S2A–E). We found that a combination of BMP4 (20 ng/ml), the WNT agonist CHIR99021 (2 µM), FGF2 (40 ng/ml), and ROCK inhibitor Y-27632 (5 µM) during the first 48 h of 3D culture enhanced cell survival and promoted formation of compact cellular aggregates (Figure S2A–F). Additionally, we pulsed the cells with the ALK5 kinase inhibitor A83-01 (2 µM) to promote TE differentiation^29^ for the first 48 h of 3D culture, and removed this inhibitor after this time to avoid a complete loss in pluripotency. Concomitantly, the concentration of FGF2 was decreased by half (20 ng/ml) for the same purpose^19^. Using this optimized condition, we observed the emergence of cavitated structures, 3 to 4 days after cell seeding (Fig. 1D). By day 6 of 3D culture, the structures exhibited a blastocyst-like morphology, forming a cohesive single outside layer, with an enlarged cavity, and an internal acentric compartment (Fig. 1E), of which 7.2% expressed the markers of the three blastocyst lineages, as judged by immunofluorescence analysis of selected markers (Fig. 1F–H). The average cell number and diameter of these hEP-structures were comparable to those of human blastocysts^31^ (Fig. 1I, J).

### EPSC aggregates bear similarities to early human embryo

**Fig. 2:**
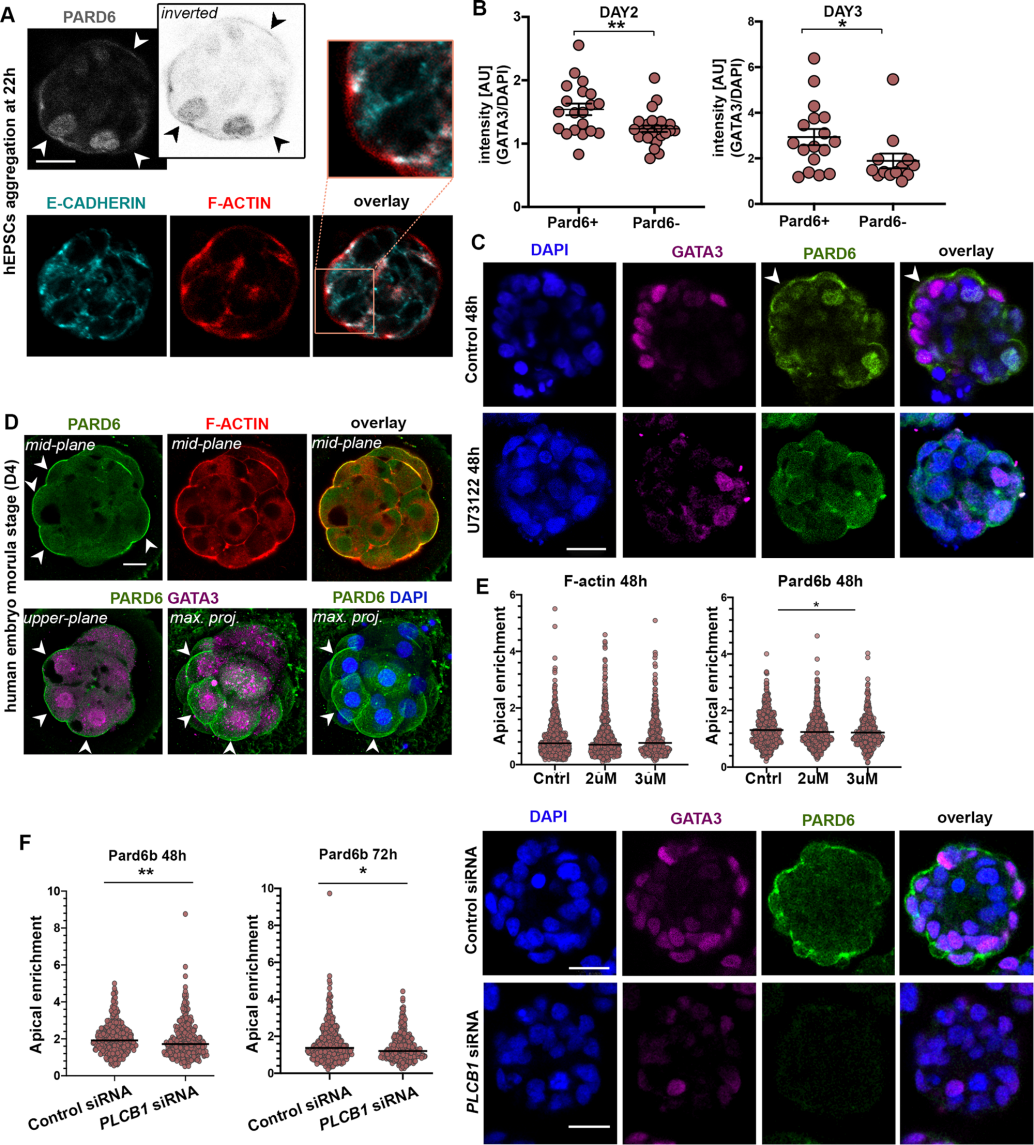
hEPSC aggregates show similarities to pre-Implantation embryo development. **A** Immunostaining of hEPSC aggregates at 22 h for PARD6 (grey), F-ACTIN (red), and E-CADHERIN (cyan). *n* = 300 aggregates, 3 experiments. **B** Quantification of GATA3 expression in cells with or without PARD6 apical enrichment observed in cells within Day 2 and Day 3 of multicellular aggregates. All measurements normalized to DAPI. Two-sided Student’s t-test; *p* = 0.0033 for Day 2; *p* = 0.0433 for Day 3; 3 experiments. Error bars represent S.E.M. **C** Immunostaining of control and U73122-treated hEPSC aggregates at 48 h for PARD6 (green) and GATA3 (magenta). *n* = 300 aggregates, 3 experiments. **D** A representative natural human embryo at morula stage (D4) stained for PARD6 (green), F-ACTIN (red), GATA3 (magenta). White arrowheads indicate apical PARD6 enrichment in the polarised cells with nuclear GATA3 expression. DAPI is shown in blue. **E** Apical enrichment quantification of F-ACTIN and PARD6b at 48 h in multicellular structures with or without addition of PLC inhibitor (U73122). Control groups received no inhibitor, while the two experimental groups were treated with either 2 uM or 3 uM U73122. Each dot represents one analysed cell. *p* = 0.0333, Kruskal-Wallis test with Dunn’s multiple comparisons test. Data is shown as mean S.E.M. *n* = 3 experiments. Also see Extended Data Fig. 3b. **F** Left: Quantification of Pard6b apical enrichment at 48 h (*p* = 0.006) and 72 h (*p* = 0.0227) in structures treated with either control siRNA or *PLCB1* siRNA. Each dot represents one analysed cell. Two-sided Mann-Whitney test. Data is shown as mean S.E.M. *n* = 3 experiments. Right: Immunostaining of GATA3 (magenta) and PARD6 (green) in structures treated with either control siRNA (top) or *PLCB1* siRNA (bottom). DAPI is shown in blue. All scale bars in the figure indicate 20 um.

The first lineage segregation event begins with compaction and cell polarisation in the mouse embryo at the 8-cell stage^32^. Only recently have studies begun to shed light on these events in human embryogenesis^33,34^. Hence, we utilised our platform to analyse the establishment and dynamics of cell polarisation at the early timepoints of multicellular aggregate formation. We observed the assembly of intercellular junctions, characterised by basolateral localization of E-CADHERIN (Fig. 2A). At the apical surface, we found distinct enrichment of F-ACTIN and PARD6 within the first 48 h of cell aggregation (Fig. 2A), indicative of cell polarisation in hEP-structures. Next, we analysed spatiotemporal expression of the transcription factor GATA3, as a marker of TE specification in human embryogenesis. GATA3 was present in the nucleus within both polarised and non-polarised cells at day 2 and day 3 of 3D culture, although its intensity was significantly higher in polarised cells showing apical enrichment of PARD6 (Fig. 2A, B). These findings correlate with observations on natural human embryos at the morula stage (Fig. 2C).

We have recently shown that the PLC-Protein Kinase C (PKC) pathway controls cellular polarisation at early stages of mouse embryo development^35^. We, therefore, treated our hEPSC 3D cultures with 2 μM and 3 μM of the PLC inhibitor, U73122. PLC inhibition resulted in a reduction of the nuclear GATA3 signal intensity (Fig. 2D, E, S3A), which correlated with a decrease in the apical enrichment of PARD6 (Fig. 2D, E, S3B). To confirm the relationship between polarisation and outer cell commitment, we next used siRNA transfection to knockdown (KD) *PLCB1* in order to deplete PLC activity in cells during 3D aggregation (Fig. 2F). The depletion of PLC activity was confirmed by qRT-PCR that RNAi depletion of *PLCB1* was effective (Figure S3C). In agreement with the previous data, we found a significant reduction in both PARD6 and GATA3 expression in hEPSC aggregates at day 3 (Fig. 2F). Thus, our results may suggest a role for the acquisition of apicobasal polarity in promoting the expression and nuclear localisation of GATA3 to drive TE specification during development of hEP-structures.

### Differentiation into embryonic and extra-embryonic lineages

**Fig. 3:**
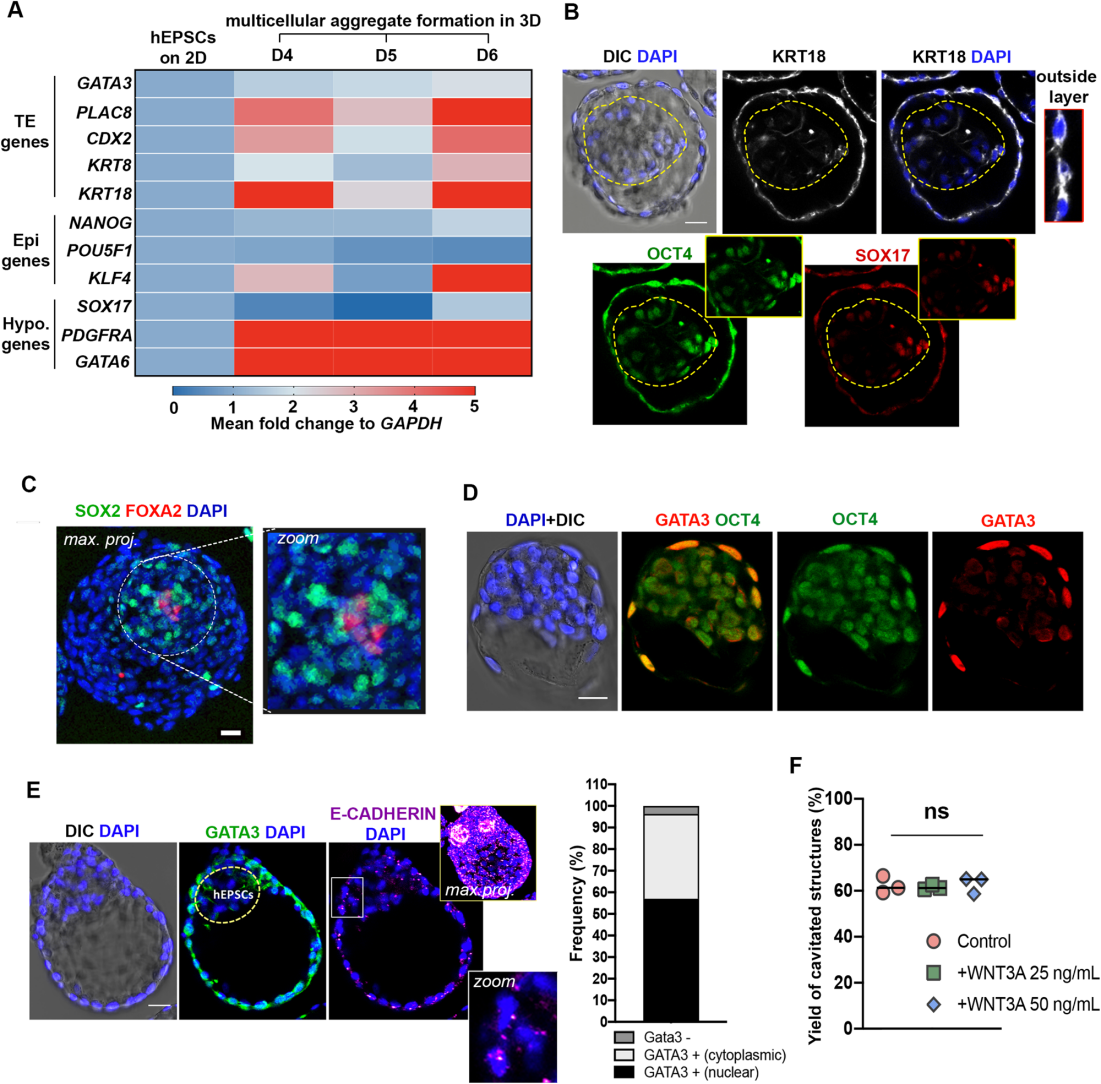
Specification of blastocyst lineages. **A** Bulk qRT-PCR analysis of blastocyst lineage marker genes in EPSCs in 2D, and multicellular aggregates at day 4, 5, 6 formed in 3D represented as a heatmap of global ΔΔCt (fold-change) to *GAPDH*. 20 cystic structures were pooled per group from 3D culture and a minimum of 10 K hEPSCs were collected from the 2D culture. **B** Immunofluorescence staining of structures generated from hEPSCs at day 5 for OCT4 (green), KRT18 (white) and SOX17 (red). Zoom image on the right shows cells with KRT18 expression. DAPI is shown in blue. n = 30 structures, 3 experiments. **C** A representative structures generated from hEPSCs at day 5 stained for SOX2 (green) and FOXA2 (red) to suggest Epi/Hypo-like inner compartment (zoom on the right). Image presented as maximum projection. *n* = 20 structures, 2 experiments. **D** Immunofluorescence staining of structures generated from hEPSCs at day 4 for OCT4 (green) and GATA3 (red). DAPI is shown in blue. *n* = 10/23 structures, 2 experiments. **E Left:** Immunofluorescence staining of GATA3 (green) and E-CADHERIN (magenta) in a representative structure at day 6. **Right:** Quantification shows frequency of structures at day 6 of 3D culture showing Gata3 nuclear expression (57.03%, 77/135 structures scored); Gata3 cytoplasmic expression (39.25%, 53/135 structures scored); no detectable Gata3 expression (3.70%, 5/135 structures scored). **F** Quantification shows frequency of cavitated structures in control and WNT3A-supplemented culture. WNT3A is applied in either at 25 or 50 ng/mL concentration. One-way ANOVA with multiple comparisons, p = 0.8473. ns, not significant. *n* = 450 for control; *n* = 483 for 25 ng/mL WNT3A; *n* = 419 for 50 ng/mL WNT3A (see source data). 3 independent experiments. Error bars show S.E.M. All scale bars in the figure indicate 20 um.

We next sought to investigate the formation of the blastocyst lineages upon cavitation of hEP-structures from day 4 onwards. We first used qRT-PCR to examine the expression level of core factors involved in establishing human blastocyst-like lineage identity (Fig. 3A). This analysis revealed that genes involved in TE specification, including *PLAC8, CDX2, KRT8,* and *KRT18*, were induced upon formation of cystic structures although *GATA3* showed only a marginal increase compared to other molecular determinants of TE identity (Fig. 3A). As expected, crucial transcription factors required for pluripotent EPI specification, including *NANOG* and *POU5F1*, showed similar levels of expression in cystic structures as in hEPSCs cultured in 2D, whereas *KLF4* was significantly upregulated in the cystic structures (Fig. 3A). Finally, we found that the expression of the core HYPO lineage determinant genes, *PDGFRA* and *GATA6*, were highly enriched in cystic structures although *SOX17* did not follow this trend (Fig. 3A).

In order to confirm these results spatially and on a protein level, we performed immunofluorescence analysis with some well-known lineage markers. In accordance with the findings from qRT-PCR, we observed enrichment for KRT18 in the outside, and expression of OCT4/SOX17 in the inner compartment (Fig. 3B). We further observed specification of the inner compartment with a second set of markers, SOX2/FOXA2^36^ (Fig. 3C). At day 4, some structures displayed constitutive expression of GATA3 in the outer cell layer while maintaining expression of the hPSC/EPI marker OCT4 (10/23 structures scored) (Fig. 3D). At later time-points in culture (Day 6, see Materials and Methods), some structures maintained GATA3 expression in the outside layer, although this enrichment became mostly cytosolic rather than nuclear (53/135 structures scored) (Fig. 3E). These late-timepoint structures also showed poor expression of E-CADHERIN at day 6 (Fig. 3E). This result likely indicates a deficiency in junction assembly during the late cavitation process and may explain the compromised expression of some TE-specific markers as in vitro development progresses.

It has been suggested that WNT3A supplementation promotes the cavitation and thus TE-like lineage identity in in vitro mouse blastoid formation^10^, which correlates with canonical WNT expression in the TE lineage during mouse blastocyst development^37^. When we tested this possibility, we found that addition of WNT3A to the culture media did not make any significant difference to the yield of cavitated structures at day 6 (Fig. 3F). This suggests that WNT3A may function differently in human development than in mouse, supporting previous claims^22,38^.

**Fig. 4:**
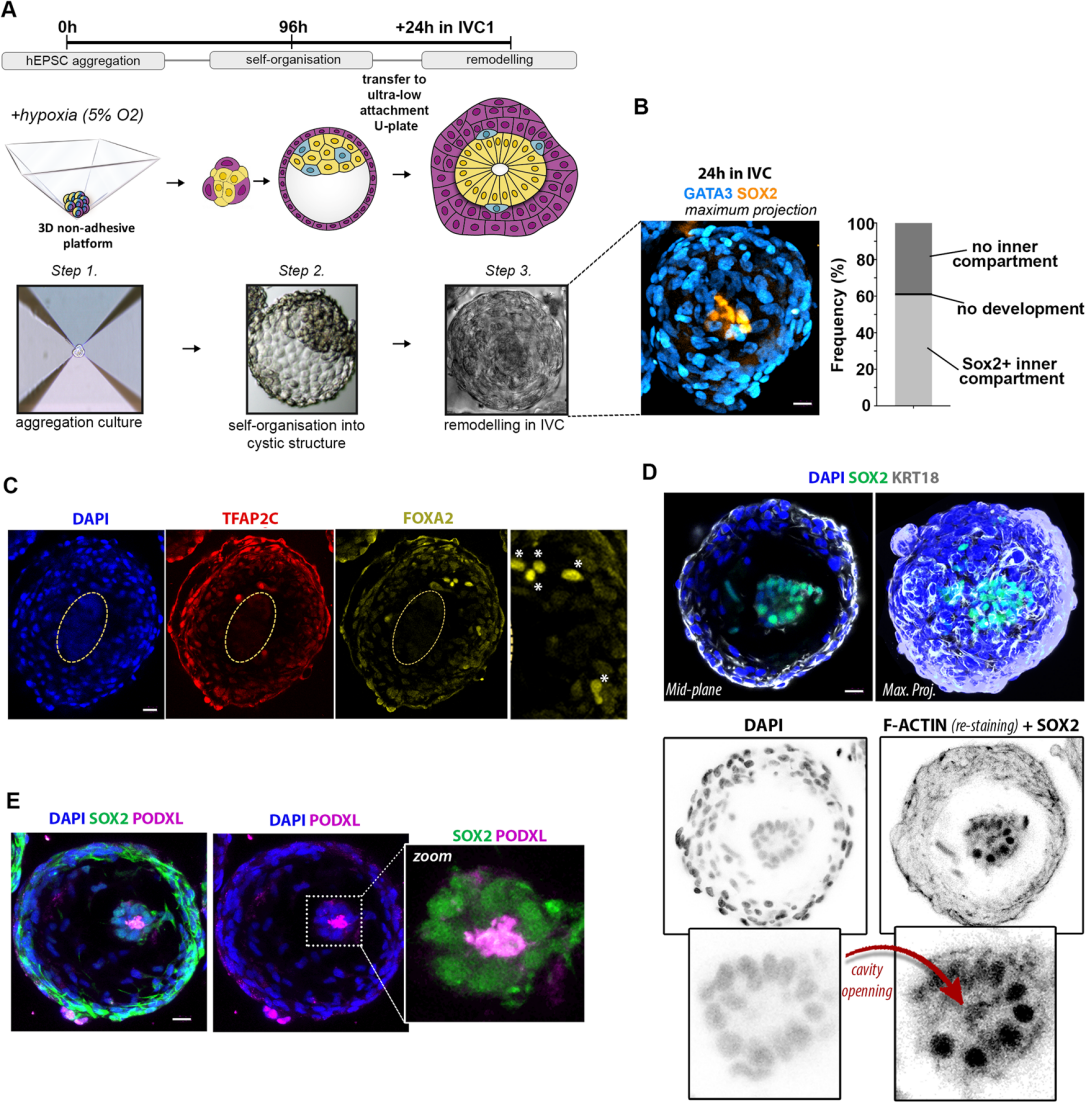
Cultured hEPSC-derived cystic structures demonstrate implantation-like morphological remodelling. **A** Illustration detailing the process of in vitro structure formation in three steps: hEPSC aggregation, self-organisation into cystic structures, and post-implantation re-organisation in IVC media (see Methods). Below are phase-contrast images showing a representative structure at each of these steps. **B** Left: Maximum projection of a representative post-implantation-like structure immunostained for GATA3 (cyan) to reveal extra-embryonic-like and SOX2 (yellow) for embryonic-like compartments. Right: quantification shows frequency of structures cultured in IVC media for 24 h that showed SOX2 + inner compartment (60.4%, light grey), no inner compartment (38.4%, dark grey), or no development (1.2%, black). *n* = 260 structures scored in 3 experiments. **C** Immunostaining showing the expression of TFAP2C (red, an extra-embryonic marker) and FOXA2 (yellow, a HYPO marker) in a post-implantation-like structure cultured in IVC for 24 h. Zoomed image on the right shows FOXA2 expressing cells. (asterisks). Yellow dashed-lines demarcates embryonic inner compartment. Representative of at least 3 independent experiments. **D** Immunostaining of post-implantation-like structure cultured in IVC media for 24 h. The top panels show a mid-plane (left) and maximum projection (right) view of a representative structure with the pluripotent compartment marked by SOX2 expression (green), surrounded by cells marked by KRT18 (white). DAPI for nuclear staining is in blue. The bottom panels show inverted images for better clarity of DAPI signal for cell nucleus on the left and F-ACTIN + SOX2 double-staining on the right. The opening of a cavity within the inner compartment (nuclear SOX2 expression) is marked by F-ACTIN. *n* = 20 structures, 3 experiments. **E** Immunostaining showing the formation of a central cavity, as marked by PODXL (magenta), within the inner compartment, marked by SOX2 (green), of an hEPSC-derived structure after 24 h culture in IVC. *n* = 20 structures, 3 experiments. DAPI staining is in blue. All scale bars in the figure indicate 20 um.

We next tested the developmental capacity of these hEP-structures to develop beyond implantation stages by culturing them in our previously established human embryo in vitro culture (IVC) platform^4^ (Fig. 4A). Within 24 h in IVC, the EP-structures reorganised into post-implantation-like morphology, 60% of which had a SOX2 positive EPI-like inner compartment surrounded by a KRT18 and GATA3 positive extra-embryonic-like compartment (Fig. 4B–D) with some structures also specifying a few FOXA2-expressing cells suggesting a HYPO-like specification (Fig. 4C). Significantly, we found that within 24 h in IVC, SOX2 positive cells in the EPI-like inner compartment became radially organised around a small central lumen (Fig. 4D). The formation of a small lumen was confirmed by PODXL expression (Fig. 4E). This indicates that hEP-structures are able to undertake some cellular rearrangements characteristic of early post-implantation human morphogenesis^3–5^.

**Fig. 5:**
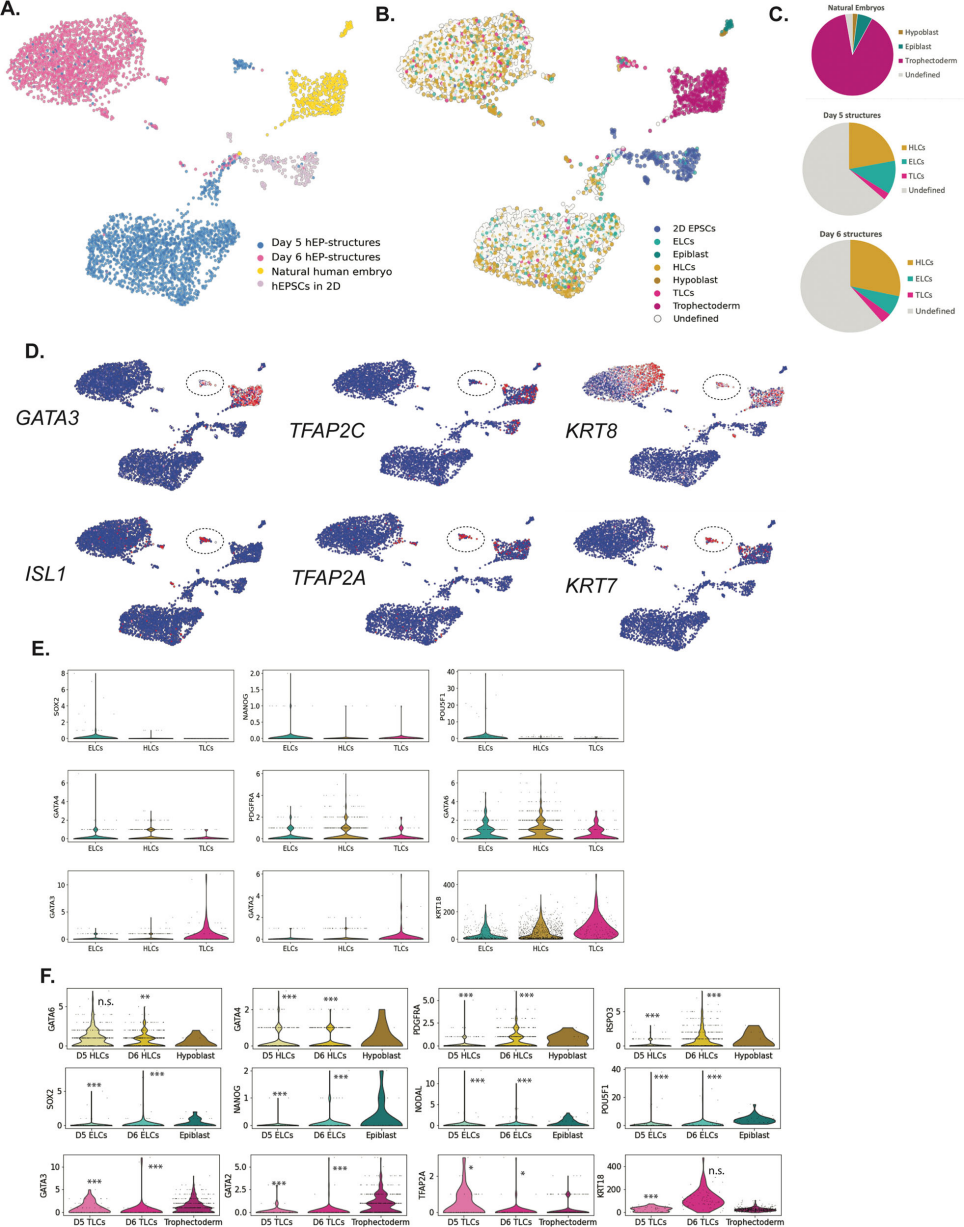
scRNA-seq analysis of hEPSC-derived cystic structures. **A** Uniform manifold approximation and projection (UMAP) grouped by cell group: natural human embryos at D5/D6 (*n* = 542 cells), hEPSCs grown in 2D (*n* = 228 cells, 2 replicates), D5 cyst structures (*n* = 2013 cells, 3 replicates), and D6 cyst structures grown in IVC for 24 h (*n* = 2057 cells, 3 replicates). **B**. UMAP showing lineage scoring of all cell groups into EPI-like cells (ELCs, teal) and EPI (dark teal), HYPO-like cells (HLCs, yellow) and Hypoblast (dark yellow), TE-like cells (TLCs, magenta), and TE (dark magenta). Undefined cells appear in grey. **C** Pie charts showing the distribution of lineage assignments for natural embryo, D5 structures, and D6 structures. **D**. Heat maps showing the expression of canonical TE markers enriched in natural cluster, as well as a subset of D5 TLCs (top row), and genes shown to be enriched exclusively in D5 TLC cluster (bottom row). A circle with a dotted line denotes D5 TLC cluster. **E** Collection of violin plots showing the relative expression of certain key lineage markers in ELCs, HLCs, and TLCs. For EPI-related genes: SOX2, NANOG, POU5F1. For HYPO-related genes: GATA4, PDGFRA, and GATA6. For TE-related genes: GATA3, GATA2, and KRT18. **F** Violin plots showing the expression of canonical markers for HYPO-related genes (yellow), EPI-related genes (teal) and TE-related genes (magenta) in Day 5 hEP-structures, D6 hEP-structures, and the natural embryo. (*p < 0.05, **p < 0.01, ***p < 0.001; (two-sided ad-hoc Dunn’s multiple comparison test applied to an ANOVA), exact p-values are listed in Supp. Data 2). For D5 structures: HLCs, *n* = 445 cells; ELCs, *n* = 235 cells; TLCs, *n* = 50 cells. For D6 structures: HLCs, *n* = 586 cells; ELCs, *n* = 144 cells; TLCs, *n* = 73 cells. For natural blastocyst, HYPO, n = 11 cells; EPI, *n* = 32 cells; TE, *n* = 484 cells. All genes were taken from Liu et al. 2021^49^, and are listed in Supp. Data 1.

Finally, in order to further characterize the transcriptional programs of our hEP-structures, we performed scRNA-seq on hEPSCs grown in 2D (prior to 3D aggregation), hEP-structures at day 5, hEP-structures grown in IVC for 24 h, and natural human blastocysts at day 5/6. Cells clustered predominantly based on sample identity (Fig. 5A, B). We assigned a lineage score based on a list of well-defined lineage markers (Supplementary Data 1) and we observed signatures of EPI-Like Cells (ELCs), TE-Like Cells (TLCs) and HYPO-Like Cells (HLCs). However, these lineages fail to cluster in UMAP space. This analysis revealed that the hEP-structures were composed of a large portion of undefined cells (Fig. 5C), similar to two other 3D models of the human blastocyst^20,21^. In addition, we also found that there was an overrepresentation of HLCs compared to HYPO cells in the natural blastocyst (Fig. 5B, C). The hEPSCs grown in 2D showed high expression of pluripotency markers such as *TDGF1*, *NODAL*, and *POU5F1*, as expected (Figure S4A) but there was considerable heterogeneity between cells. In day 5 hEP-structures we found a small subpopulation of *GATA3*-positive cells, which clustered close to the TE cluster of the natural embryo (Fig. 5D). This subcluster also expressed several amnion markers, such as *ISL1*, suggesting that it may share properties with the amnion rather than TE as has been reported in iBlastoids derived from reprogrammed hiPSCs^39^ (Fig. 5D). Overall, while we do see expression of some key markers in our ELCs, HLCs and TLCs (Fig. 5E), there was a disproportionate representation of lineages in our hEP-structures as seen by the overrepresentation of HYPO-specific markers, with relatively little expression of TE-specific markers.

To better understand how the hEP-structures compared to natural human blastocysts at day 6, we compared the expression patterns of the key marker genes used to define the ELC, HLC and TLC signatures (Fig. 5F, S5A–C, see Supplementary Data 2). This revealed that over half of the genes (52/96 at day 5, 50/96 at day 6) did not differ between ELCs and the human EPI, and a similar number of genes were expressed at comparable levels between the HLCs and HYPO (52/96 and 53/96). Interestingly, of those genes upregulated in HLCs compared to the HYPO, several were extracellular matrix (ECM) proteins including *COL4A1, COL18A1*, and *FN1*. Importantly, these ECM genes were aberrantly expressed in ELCs and TLCs as well (Figure S5D). In the TLCs, there was an observable shift between day 5 and day 6 in this signature where 43/96 genes were expressed at a similar level to the blastocyst TE at day 5, which decreased to 25/96 at day 6 (Fig. 5F, S5C, see Supplementary Data 2). In order to take a more global view of the differences between the hEP-structures and the natural blastocysts, we performed an unbiased comparative analysis of gene programs. This aims to identify clusters of cells which may upregulate blocks of gene programs. This revealed several gene programs upregulated in either the hEP-structures or natural blastocysts. These included ECM genes *COL3A1*, *COL4A2, COL4A1, COL1A1*, *COL6A3*, and *FN1*. Additionally, some genes purportedly related to the amnion—though also expressed in the TE—such as *POSTN* and *TPM1* were enriched in hEP-structures (Figure S6A). These hEP-structures and natural blastocyst enriched genes were subjected to a simple gene set enrichment analysis using Reactome gene sets broadly related to signalling activities. This revealed an enrichment of several terms, including those related to PDGF, Interleukin, VEGF, PI3K, STAT3, and WNT signalling for the hEP-structures (Figure S6B).

Taken together, we conclude that although the transcriptional machinery for blastocyst lineage programming appears to be initiated at some level in hEP-structures in our protocol, a continuum of cell fates then develops, indicating that complete trans-differentiation is not attained.

### Co-assembly of EPSCs and human trophoblast stem cells

The restricted specification of the TE-like lineage and potential questions regarding the differentiation of TE versus amnion fate in these structures generated solely from hEPSCs led us to question whether these cells possess the capacity to generate the TE-like lineage. We were recently able to correct a similar shortcoming in mouse blastoids by combining EPSCs with mouse trophoblast stem cells (TSCs)^11^. As a recent study has shown it is possible to derive human TSCs from first trimester human placental samples and blastocysts^29^, we asked whether such TSCs could participate alongside hEPSCs in the assembly of human blastoids (Figure S7A). To this end, we first allowed hEPSCs to aggregate for 24 h after which we added hTSCs^11^. The first 24 h of hEPSC aggregation demonstrated the increased potential to generate both embryonic EPI-like and extra-embryonic HYPO-like lineages in the EP condition compared to the Rset condition that has been shown to promote naïve pluripotency^40^ (Figure S7B). Within 4 days of co-culture, we observed the formation of cystic structures having internal acentric hEPSC compartments. We also observed robust expression of some of the blastocyst lineage markers, including GATA3, SOX2, and SOX17 (Figure S7C). Nevertheless, it appeared that these structures struggled to form a cohesive TE-like epithelium and displayed not one but multiple cavities (Figure S7C). Thus, in contrast to our experience with mouse TSCs, this human TSC line appears unable to rescue the shortcomings of human ESPCs to generate the TE lineage. Thus, future work will be required to generate human TSC lines having the ability to recapitulate pre-implantation development in order to enhance this system further.

## Discussion

Cultured pluripotent stem cells show a dynamic spectrum of pluripotency states, reflecting stages in pre-to-post-implantation development in vivo, principally the transition from the naïve to the primed state^41–43^. This dynamic spectrum of pluripotency states exhibits distinct molecular and functional properties that affect the differentiation potential of cells and their ability to contribute to chimera formation^27,44^. Previous reports have demonstrated the ability of mouse and human EPSCs to contribute to chimeras^25^ and show integration into both embryonic and extra-embryonic parts, a property not seen with other PSCs^25,27^. Thus, in the current study, we aimed to leverage this reported bi-potency of hEPSCs by developing a strategy enabling us to reconstitute the spatiotemporal lineage differentiation and self-organisation of human early development. We assessed the multi-differentiation potential of hEPSCs to model early embryonic cell fate in a 3D culture platform. The results we present show that the hEPSC-based system we have established here allows hEPSCs to self-organize into structures that bear some resemblance to human blastocysts and peri-implantation stage embryos in morphology and, to a certain extent, also in lineage specification. However, we also show that their cell-lineage composition is imperfect, and these cells mainly adopt an intermediate transcriptional state.

Our knowledge of how to capture stem cell pluripotency in vitro is rapidly evolving and recent reports have challenged the previously proposed bi-potency of EPSCs^44,45^. Consistent with these reports, our previous study suggested that mouse EPSCs could not generate TE-like progeny in mouse blastoids, at least in our hands^11^. It has recently been reported that both mouse and human EPSCs are more similar to early post-implantation EPI cells and that the differentiation of pluripotent cells in either the naive or primed state may proceed toward different fate trajectories, suggesting that cells in different states might respond differently to stimuli^27,44,45^. Consistent with these observations, we find that hEPSCs mainly express genes that are specific to the late stage epiblast. We also observe that hEPSCs can adopt different morphologies under routine culture with some cells displaying features characteristic of pluripotent cells in the naïve state and others in the primed state, in different ratios after each passage. This possibly reflects the range of developmental potencies associated with the hEPSCs we demonstrate here. However, while cells of the structures developing in our study display many EPI-like and HYPO-like markers and some TE-like markers, a full range of lineage markers is not expressed, and many of these markers are expressed at levels different from the *bone fide* blastocyst lineages. This is in line with recent findings emphasizing the restricted potency of mouse EPSCs^45^. Together, our results suggest important limitations in the molecular and epigenetic plasticity of hEPSC-derived structures leading to weak activation of important genes such as *GATA3*, *SOX2*, and *SOX17* resulting in inefficient lineage specification.

In our study to generate mouse blastoids, we had been able to correct some of the deficiencies of mouse EPSCs by co-culturing them with TSCs^11^. However, our attempts using a human TSC-line were not similarly encouraging. Given that these hTSCs are reported to be most similar to villous cytotrophoblasts^29^, it is likely that these hTSCs may be more conducive to post- rather than pre-implantation development.

Recently alternative methods to generate human blastocyst-like structures have been described using naïve or induced pluripotent human stem cells^20,21,46^. In these reports, the resulting structures recapitulate the overall morphology of the blastocyst, with an inner cell mass and blastocoel cavity, similar to the structures presented here. Formation of structures with proper architecture, with a cavity and inner cell mass, ranges from 9.4 to 12.8% for human naïve blastocyst-like structures^21^ and 5.8–18% for structures generated from iPSCs^20^. Additionally, the relationship between the efficiency for generation of proper structure morphology and correct segregation of all three lineages remains unclear and several gene programs do not appear to be shared between these models. While iPSC-derived blastoids seem to have the morphological and transcriptional organization, questions have been raised regarding the identity of their TE-like cells, as they appeared more similar to reported amnion-like cells^39^. This finding, in combination with our own, raises interesting disconnects between promising morphologies and cell behaviours in stem cell-derived models of human embryogenesis and transcriptional cell identities. This work also shows the ability to generate morphologically similar structures with drastically different gene expression patterns, highlighting the uncoupling of morphology and gene expression in these models. These are yet to be explored and overcome in future work and emphasizes the need for stringent and comprehensive analyses to better understand the functionality of these models. Nevertheless, these recent studies, together with our own, will inform efforts to enhance the efficiency of both correct morphogenesis and robust lineage segregation in human embryo models.

In summary, our findings demonstrate that hEPSCs are not the equivalent of totipotent blastomeres and they are only partially able to specify embryonic cell progeny. This may reflect distinct molecular trajectories and an intermediate state adopted by these cells that lead to the generation of the improperly differentiated cells observed in this study. Nevertheless, these cells are able to generate multicellular structures showing some of the key morphological features and aspects of patterning similar to natural early human embryos. Thus, the system we present here may offer an alternative route with the potential to be harnessed into a fully functional embryo-like platform in vitro. We anticipate that, despite these shortcomings, this system together with others recently described, has the potential to lead to a variety of future applications that will be pivotal in unravelling many of the enigmas of human developmental regulation.

## Methods

### Data reporting

No statistical methods were used to predetermine sample size. The experiments were not randomized and the investigators were not blinded to allocation during experiments and outcome assessment.

### Ethics statement

Stem cell-derived multicellular structures described in this study show no evidence of germ line patterning, thus they do not have human organismal form or potential. Additionally, all experiments were terminated by no later than day 8 in vitro. Our research was subject to review and approval from the Human Embryo and Stem Cell (HESC) Committee of California Institute of Technology, in compliance with the ISSCR 2016 guidelines. The human embryo work at California Institute of Technology was approved by the California Institute of Technology Committee for the Protection of Human Subjects (Institutional Review Board number 19-0948). Funding was obtained through Open Philanthropy Project fund at the Silicon Valley Community Foundation. Human embryos at the blastocyst stage were obtained from the University of Southern California (USC) through the preexisting USC Institutional Review Board-approved Biospecimen Repository for Reproductive Research (HS-15-00859) after appropriate approval was obtained unanimously from the Biorepository Ethics Committee.

### Human embryo thawing

The human embryos at the blastocyst stage were warmed using Embryo Thaw Media Kit following the manufacturer’s instructions (Fujifilm Irvine Scientific, Cat. No. 90124). The day before warming, Continuous Single Culture-NX Complete medium (Fujifilm Irvine Scientific, Catalog No: 90168) was equilibrated overnight at 37 °C + 5% CO_2_. On the day of warming (day 1), the straw that contains the embryo was defrosted at room temperature for 30 s and immersed in prewarmed (37 °C) water for 1 min until ice melted. The embryo was then transferred into T-1 (5 min), T-2 (5 min), T-3 (10 min) solutions for slow warming and finally into Multipurpose Handling Medium (MHM, Fujifilm Irvine Scientific, Cat. No. 90163) for recovery. All these incubation steps were done using 4 well plates (Nunc) and 1 ml per solution. Warmed embryos were finally incubated in drops of preequilibrated Continuous Single Culture-NX Complete medium under mineral oil (9305, Irvine Scientific). Culture conditions are the following: 37 °C 21% O_2_ and 5% CO_2_. Embryos were incubated for a total of 24 h until used for further RNA-sequencing protocols.

### Human cell lines

The hPSC lines utilized in this study include: RUES2-GLR (kindly provided by Ali Brivanlou, The Rockefeller University, US), and ESI017 (kindly provided by Michael Elowitz, California Institute of Technology, US). Human TSCs (TS^CT^) were kindly provided by Hiroaki Okae and Takahiro Arima (Tohoku University Graduate School of Medicine, Japan). Each of these cell lines was tested negative for mycoplasma contamination, which was monitored on a bi-monthly basis (MycoScope™ PCR Mycoplasma Detection Kit, Genlantis).

### Cell culture

All hEPSC lines were maintained under 20% O_2_ and 5% CO_2_ at 37 °C conditions on irradiated CF1 mouse embryonic fibroblasts (MEF) feeder cells. hEPSCs were grown using ‘human Expanded Potential’ (hEP) medium consisting of DMEM/F12 (Thermo Fisher Scientific, 11320-033), Neurobasal-A (Thermo Fisher Scientific, 21103-049), N2 supplement (Thermo Fisher Scientific, 17502-048), B27 supplement (Thermo Fisher Scientific, 12587-010), 1% GlutaMAX (Thermo Fisher Scientific, 35050-061), 1% nonessential amino acids (Thermo Fisher Scientific, 11140-050), 0.1 mM b-mercaptoethanol (Thermo Fisher Scientific, 31350-010), penicillin-streptomycin (Thermo FisherScientific, 15140–122) and 5% knockout serum replacement (KSR, Thermo Fisher Scientific, A3181502). LCDMYI supplementation was added as indicated at the following concentrations: 10 ng ml^−1^ recombinant human LIF (L, 10 ng ml^−1^; Peprotech, 300-05), CHIR99021 (C, 1 mM; Stem Cell Technologies), (S)-( +)-Dimethindenemaleate (D, 1 mM; Tocris, 1425) and Minocycline hydrochloride (M, 2 mM; Santa Cruz Biotechnology, sc-203339), 1. All hEPSCs were used before reaching P70 and cell cultures were examined by eye to monitor for spontaneous differentiation of colonies into mesenchymal-like cells.

hTSCs were cultured on 6-well plates pre-coated with 5 mg/ml Col IV at 37 C for at least one hour, as previously described in Okae et al.^29^. Cells were grown in ‘human Trophoblast stem cell’ (hTS) medium consisting of DMEM/F12 supplemented with 0.1 mM b-mercaptoethanol, 0.2% FBS, 0.5% Penicillin-Streptomycin, 0.3% BSA (A8806-5G, Sigma-Aldrich), 1% ITS-X supplement (51500-056, Thermo Fisher Scientific), 1.5 mg/ml L-ascorbic acid (A4403, Sigma-Aldrich), 50 ng/ml EGF (62253-63-8, Sigma-Aldrich), 2 mM CHIR99021, 0.5 mM, A83-01 (72024, Stemcell Technologies), 1 mM SB431542 (Stem Cell Technologies), 0.8 mM VPA (Sigma-Aldrich) and 5 mM Y27632

### Preparing and plating cell suspensions for “AggreWell” aggregation experiments

AggreWell 400 format plates were prepared following the manufacturer’s protocol. Briefly, wells were rinsed with the rinsing solution (Stem Cell Technologies), centrifuged for 5 min at 2000g and incubated at room temperature in the tissue culture hood for 20 min. The wells were then washed with 2 ml of 1× PBS. After PBS removal, 500 ml of final culture medium (*IVF-hEP-hTS*, see below) was added to each well and the plate placed at 37 C and 5% CO_2_ until ready to use.

### Generation of multicellular aggregates in 3D

To begin, hEPSCs were dissociated to single cells by incubation with Accutase (07920, Stem Cell Technologies) at 37 °C for 3 min. Cells were collected and pelleted by centrifugation for 4 min at 300 g and resuspended in hEP-LCDMYI medium (described above). This cell suspension was pre-incubated at 37 °C in an atmosphere of 5% CO_2_ on gelatinized tissue-culture-grade plates for 30 min to remove inactive MEFs.

Post incubation on gelatin plates, cells were counted using a haemocytometer and a total of 7200 hEPSCs was added to 1 mL of media composed of 50% *IVF* media (Continuous Single Culture-NX Complete (CSCM-NXC)) (90168, FUJIFILM), 25% *hEP* media, and 25% *hTS* media. This media was also supplemented with CHIR99021 (2uM), Y27632 (5uM), BMP4 (20 ng/mL), FGF2 (40 ng/mL), and A83-01 (2uM). Cell suspensions were added dropwise to the Aggrewells. All wells without cells were filled with 1 mL PBS to humidify the local atmosphere to minimize evaporation. The AggreWell plate (24-well, 1200 Aggrewell format) was then centrifuged for 3 min at 100 g, and placed at 37 °C under hypoxic conditions (5% CO_2_ and 5% O_2_). After 48 h, media was removed from wells and replaced with fresh culture media as described above, although FGF2 concentration was lowered to 20 ng/mL and A83-01 was omitted. Cells were left to grow for an additional 48-72 h until proper morphology was observed, at which point structures were fixed for immunostaining or transferred to IVC media (see below) for mimicking development beyond implantation stages.

### Criteria for selecting multicellular aggregates structures

Following completion of any given aggregation experiment (from day 4 to 6), all cystic structures those clearly displaying a cavity were included in further analyses. Non-cavitated structures were excluded from downstream analyses.

### Co-culture of hEPSCs with hTSCs

To perform two-step aggregation experiments, hEPSCs were first seeded as described above hEP-LCDMYI media. After a 24 h period of aggregation, hTSC colonies were dissociated to single cells, and counted using a haemocytometer. For aggregation experiments, 50% *hEP* media and 50% *hTS* media is used as described above. A total of 16,800 hTSCs were added per well (24-well, 1200 Aggrewell format) and the plate was placed at 37 °C, 5% CO_2_ and 5% O_2_.

### In Vitro Culture (IVC) of hEPSC-derived structures

To prepare plate for in vitro culture, 150 µL of modified *IVC1* (*mIVC1*) media was added to each well of a 96-well ultra-low attachment U-shaped plate (7007, Costar). *mIVC1* media consisted of the following: Advanced DMEM/F12 (12634-010; Thermo Fischer Scientific; Waltham, US) supplemented with 20% (vol/vol) heat-inactivated FBS (16141079, Thermo Fisher Scientific), 2 mM GlutaMAX, penicillin (25 units/ml)/Streptomycin (25 μg/ml), 1X ITS-X (10 mg/L insulin, 5.5 mg/L transferrin, 0.0067 mg/L sodium selenite, 2 mg/L etholamine; 51500-056; Thermo Fisher Scientific; Waltham, US), 8 nM β-estradiol (E8875; Sigma-Aldrich; St. Louis, US), 200 ng/ml progesterone (P0130; Sigma-Aldrich; St. Louis, US), 25 μM *N*-acetyl-_L_-cysteine (A7250; Sigma-Aldrich; St. Louis, US), 17 nm IGF1, 20 ng/mL FGF2 (Gibco), FGF4 (25 ng/mL; R&D Systems, 5846-F4) and heparin (1 mg ml^-1^; Sigma, H3149).

### Immunofluorescence staining

Stem cell-derived structures were fixed in 4% paraformaldehyde (Electron Microscopy Sciences, 15710) for 20 min at room temperature, and then washed twice in PBT [phosphate-buffered saline (PBS) plus 0.05% Tween-20]. Structures were permeabilized for 30 min at room temperature in PBS containing 0.3% Triton-X-100 and 0.1% glycine. Primary antibody incubation was performed overnight at 4 °C in blocking buffer [PBS containing 10% fetal bovine serum (FBS), 1% Tween-20]. The following day, embryos were washed twice in PBT, then incubated overnight at 4 °C with secondary antibody (1:500) in blocking buffer. Structures were washed twice in PBT buffer and then transferred to PBT drops in oil-filled optical plates before confocal imaging. The antibodies used are given in Supplementary Table 1.

For human embryo images shown in Fig. 2e, embryos were fixed in IVIRMA Valencia, washed twice in a PBS solution containing 0.1% Tween-20 (Sigma, cat. no. P9416) and immediately placed into a 0.5 ml PCR tube within an oil-PBS-oil interphase. Tubes were stored at 4 °C were shipped to the University of Cambridge for immunofluorescence.

### Image data acquisition, processing, and quantification

Fluorescence images were acquired with an inverted Leica SP8 confocal microscope (Leica Microsystems), using a Leica Fluotar VISIR 0.95 NA 25x objective. Fluorophores were excited with a 405-nm diode laser (DAPI), a 488-nm argon laser (GFP), a 543-nm HeNe laser (Alexa Fluor-543/555) and a 633-nm HeNe laser (Alexa Fluor-633/647). Images were acquired with 0.5–1.2 mm z-separation. Raw data were processed using open-source image analysis software Fiji Image J (version: 2.0.1) open access software and assembled in Photoshop CC 2019 (Adobe). Digital quantifications and immunofluorescence signal intensity graphs were obtained using Fiji software^47^.

*Apical enrichment analysis:* F-actin and PARD6 polarisation were measured in a single focal plane, by taking the middle plane of the aggregate. A freehand line of the width of 0.5μm was drawn along the cell-contact free surface (apical domain), or cell-contact (basal) area of the cell, signal intensity was obtained via the Region of Interest (ROI) function of Fiji. The apical/basal signal intensity ratio is calculated as: I(apical)/I(basal). A cell is defined as polarised when the ratio between the apical membrane and the cytoplasm signal intensity exceeds 1.5.

*GATA3 expression analysis:* the nucleus of each cells is masked using the Region of Interest (ROI) tool of Fiji. The average signal intensity of the ROI is calculated and a cell is defined as GATA3 positive when the nucleus to cytoplasm signal intensity exceeds 1.5.

### siRNA-mediated knock-down in hEPSC-derived aggregates

Transfections of siRNA were performed using Lipofectamine RNAi MAX (13778075, Thermo Fisher Scientific) according to the manufacturer’s instructions. Upon seeding hEPSCs into AggreWells (as described above), Lipofectamine and siRNA (Qiagen, Hs_PLCB1_4, SI00115521; Qiagen, Hs_PLCB1_6, SI02781184; Qiagen, negative control siRNA, 1022076) against target genes with Opti-MEM (31985070, Gibco) is mixed and the mixture of either control siRNA or PLCB1 siRNA were evenly added into each well. Cell aggregates at 48 h were collected to analyse the gene expression by qRT-PCR.

### Bulk qRT-PCR analysis

Total RNA was extracted with using Arcturus PicoPure™ RNA Isolation Kit (12204-01, Applied Biosystems) as per manufacturer’s instructions. QRT-PCR was performed with the Power SYBR Green RNA-to-CT 1-Step Kit (Life Technologies) and a Step One Plus Real-time PCR machine (Applied Biosystems). The amounts of mRNA were measured with SYBR Green PCR Master Mix (Ambion). Relative levels of transcript expression were assessed by the ∆∆Ct method, with Gapdh as an endogenous control. For qPCR primers used, see Supplementary Table 2.

### Single cell isolation of in vitro cultured human embryos and hEPSC-derived aggregates

Human blastocyst (*n* = 6) were exposed to Tryple Express Select ×10 (ThermoFisher A1217701) for 15 min in 37 °C, and subsequently dissected with glass capillaries of different diameters.

hEPSC-derived structures were cultured until Day 5 (see Generation of multicellular aggregates in 3D) or Day 6 (see In Vitro Culture of hEP-structures beyond implantation). We then selected structures based on the morphological criteria of having a cavity and acentric compact inner cell mass with glass capillaries. Roughly 50 structures were collected for Day 5 and Day 6 and each condition was performed in triplicate, for a total of ~150 structures per condition. hEP-structures were then first exposed to Tryple Express Select ×10 (ThermoFisher A1217701) for 15 mins in 37 °C, and subsequently dissected with glass capillaries of different diameters.

### Single cell isolation of hEPSCs in 2D culture

hEPSCs were washed once with PBS and dissociated with Accutase (07920, Stem Cell Technologies) at 37 °C for 3 min. Cells were collected and pelleted by centrifugation for 4 min at 300 g and resuspended in hEP-LCDMYI medium (described above). This cell suspension was pre-incubated at 37 °C in an atmosphere of 5% CO_2_ on gelatinized tissue-culture-grade plates for 30 min to remove inactive MEFs.

### Single-cell mRNA-sequencing

For single-cell sequencing, we used lipid-modified oligonucleotides (LMOs)^48^ to multiplex multiple samples into a single droplet microfluidics run. Dissociated cells from Day 5 hEP-derived structures, Day 6 hEP-derived structures, and 2D hEPSCs were labelled with sample-specific lipid-modified oligos (LMOs). Samples were washed to remove any leftover LMOs and then pooled into a multiplexed cell suspension that was run on a single lane of a 10× Genomics chip, using v3.0 reagents. Cells from the natural embryo were not multiplexed but were run concurrently on a parallel lane within the chip. The single-cell sequencing library was prepared as per the manufacturer’s instructions and sequenced on an Illumina Hiseq 4000 at a minimum coverage of 20,000 PE reads per cell (read 1: 28 bp, i7: 8 bp, read 2: 91 bp). Sample-specific LMO tags were separately amplified according to Ding et al.^49^ and sequenced at a read depth of 2,016 reads per cell. Sample demultiplexing was performed using an in-house demultiplexing pipeline that discovers sample-specific thresholds^50^, scores each cell as being positive or negative for a specific tag, and retains only singly-labelled cells.

### Single cell RNA-seq data analysis

Single-cell RNA-sequencing was performed using the 10× Genomics Chromium system. Reads were aligned against GRCh38. Further downstream analyses were performed in Python using the Scanpy toolkit (version 1.7.2) and Anndata (version 0.7.5). No cells were filtered for mitochondrial or ribosomal content. Initial analysis including normalization, scaling, identification of highly variable genes, and clustering was performed as described in the kalisto | bustools tutorial “Introduction to single-cell RNA-seq II: getting started with analysis”^51,52^. Single-cell data was further visualized using the UMAP dimensionality reduction, as determined by the sc.tl.umap function in Scanpy. Lineages were defined using sc.tl.score_genes function in scanpy and the gene list used for this function were taken from Supplementary Table 12 of Liu et al. (type: ALL-TE, ALL-EPI, ALL-PE)^53^, and are included as Supp. Data 1. A score for each lineage was given to every cell, and the “lineage” designation for each cell was determined by the highest score of the 3. Any cells with scores below 0.08 were denoted as “undefined”. All violin plots were made using sc.pl.violin function in Scanpy.

### Unsupervised clustering of gene expression programs

To compare global gene expression patterns across datasets, we constructed large-scale heatmaps for each dataset clustered using unsupervised methods (orthogonal non-negative matrix factorization for gene programs and hierarchical clustering for cells^54^). First, we integrated multiple datasets together by keeping only the intersection of genes found across all datasets (*n* = 18379 genes). We then used the PopAlign framework to filter highly variable genes, and normalize the datasets. Normalization was done by dividing each transcript count by the sum total within the cell, scaling by 10,000, adding a + 1 pseudocount, and then logging. To find gene programs, we randomly sampled 5,000 cells across the integrated dataset, and then ran orthogonal non-negative matrix factorization find a set of 16 feature vectors^48^. We discovered the top genes within each feature, and used this gene list to organize the gene expression heatmaps for each dataset (popalign.plot_top_gene_features). Cells within each dataset were clustered using scipy hierarchical clustering (scipy.cluster.hierarchy) using correlation distance, and linkage = complete. Gene programs were then manually reorganized into groups which were Universal across all datasets, or more specifically enriched in the Natural Embryo, D5, or D6 stem cell-derived embryo-like structures. We then ran a gene set enrichment analysis based on the hypergeometric test (popalign.enrichment_analysis) on these gene groups using Reactome-signalling gene sets to identify specific signalling pathways that are up-regulated.

### Statistics and reproducibility

Statistical tests were performed on GraphPad Prism 8.4.3 software. Figure legends indicate the statistical tests used and number of independent experiments performed in each analysis. All error bars defined in the legends. Unless otherwise noted, each experiment was performed at least two times. Statistical significance: *p* < 0.05 was considered statistically significant (*), *p* < 0.01 (**), *p* < 0.001 (***), *p* < 0.0001 (****).

### Reporting summary

Further information on research design is available in the Nature Research Reporting Summary linked to this article.

## Supplementary information

Supplementary Information

Reporting Summary

Description of Additional Supplementary Files

Supplementary Data 1

Supplementary Data 2

## Data Availability

The scRNA-seq data for 2D hEPSCs, hEP-structures, and natural human blastocyst generated in this study have been deposited in the GEO database under accession code GSE178326 [GEO]. Published iBlastoid and StemBlastoid datasets used in this study were obtained from Liu et al.^20^ and Yu et al.^21^ under accession numbers GSE156596 and GSE150578, respectively. Source data are provided with this paper.

## Source data

Source Data

## Code availability

The code generated in this study is provided at https://github.com/vjorgensen/hEP-structures_MZG.
