# Supplementary material for: Reconstructing aspects of human embryogenesis with pluripotent stem cells
Source: Nat Commun. 2021 Sep 21;12:5550. doi: 10.1038/s41467-021-25853-4 (PMC8455697; doi:10.1038/s41467-021-25853-4)
Supplement: Supplementary file 1 — Supplementary Information [file 41467_2021_25853_MOESM1_ESM.pdf]

Supplementary Figure 1

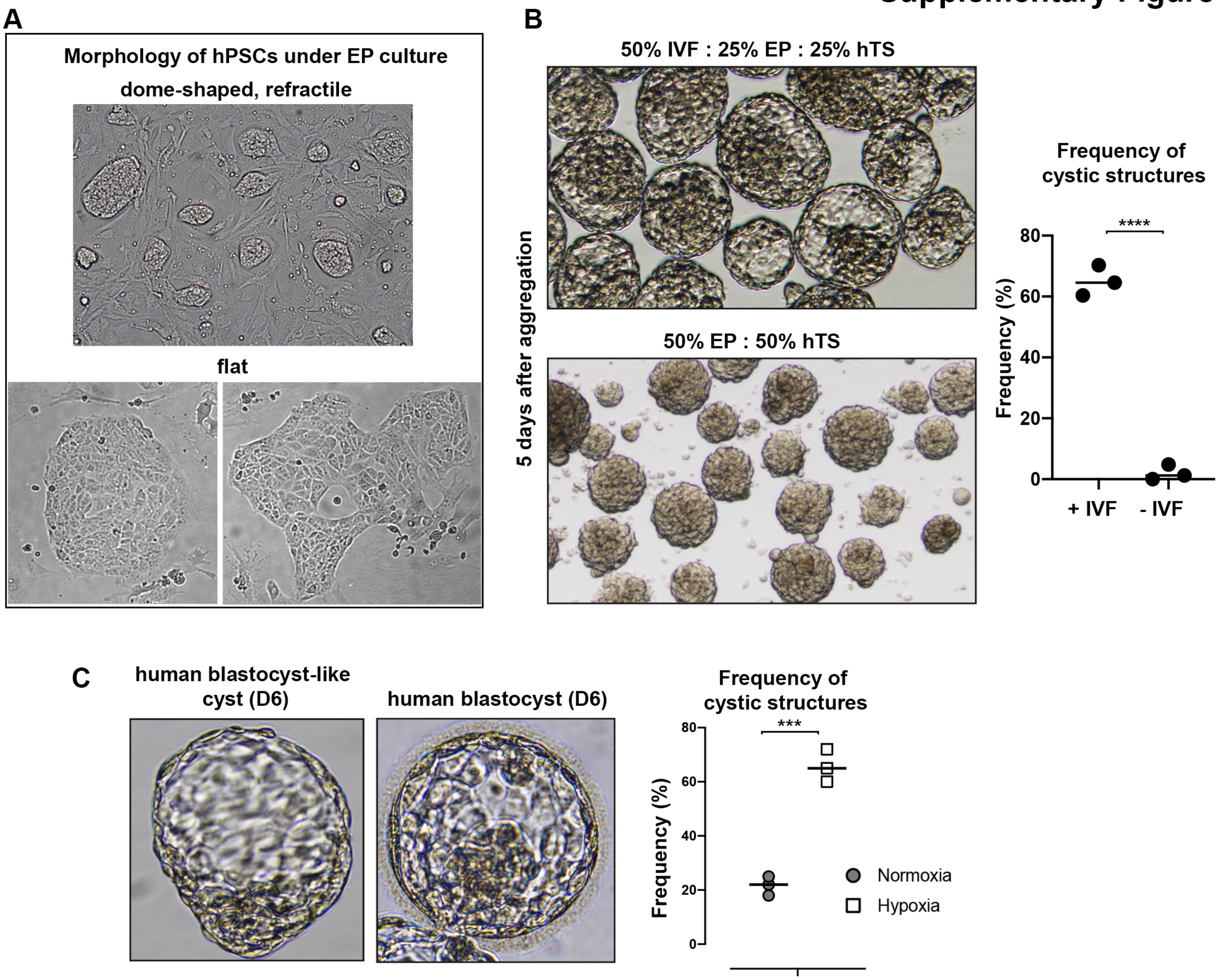

Supplementary Figure 1. Characterisation of cellular and structural morphologies under 2D and 3D culture conditions. A. DIC images showing varied colony morphology of hPSCs under EP culture conditions. The top image shows dome-shaped morphology, characteristic of pluripotent cells at the naïve state, while the bottom images show flat colonies, characteristic of cells in the primed state. Representative of at least 10 independent experiments. **B.** DIC images of hEP-structures grown for 5 days with (top) and without (bottom) IVF media. Quantification on the right shows the frequency of cystic structure formation in each condition (+/- IVF). Two-sided Student's t-test; ****$p<0.001$; 3 experiments. Error bars show S.E.M. **C.** Comparison of hEP-structures at day 6 (D6) of 3D culture to the natural human blastocyst at the same developmental time point. Quantification on the right shows the frequency of cystic structure formation under 20% O_2 (Normoxia; grey circle) and 5% O_2 (Hypoxia; white square) conditions. Two-sided Student's t-test; $p=0.005$; 3 experiments. Error bars show S.E.M. All scale bars in the figure indicate 20 um.

Supplementary Figure 2

A

screening of GFs and inhibitors — features observed

	BMP4	CHIR	Y2	FGF2	A83	CELL SURVIVAL	CAVITATION	SOX2+ INNER COMPARTMENT	KRT18+ OUTER COMPARTMENT
C1	X	√	√	√	√	√	X	N/A	N/A
C2	√	X	√	√	√	X	N/A	N/A	N/A
C3	√	√	X	√	√	X	N/A	N/A	N/A
C4	√	√	√	X	√	√	X	N/A	N/A
C5	√	√	√	√	X	√	√X	N/A	X
C6	√	√	√	√	√	√	√	√	√

√ High frequency / reproducible
√X Low frequency / low reproducibility
X Compromised
N/A Not available

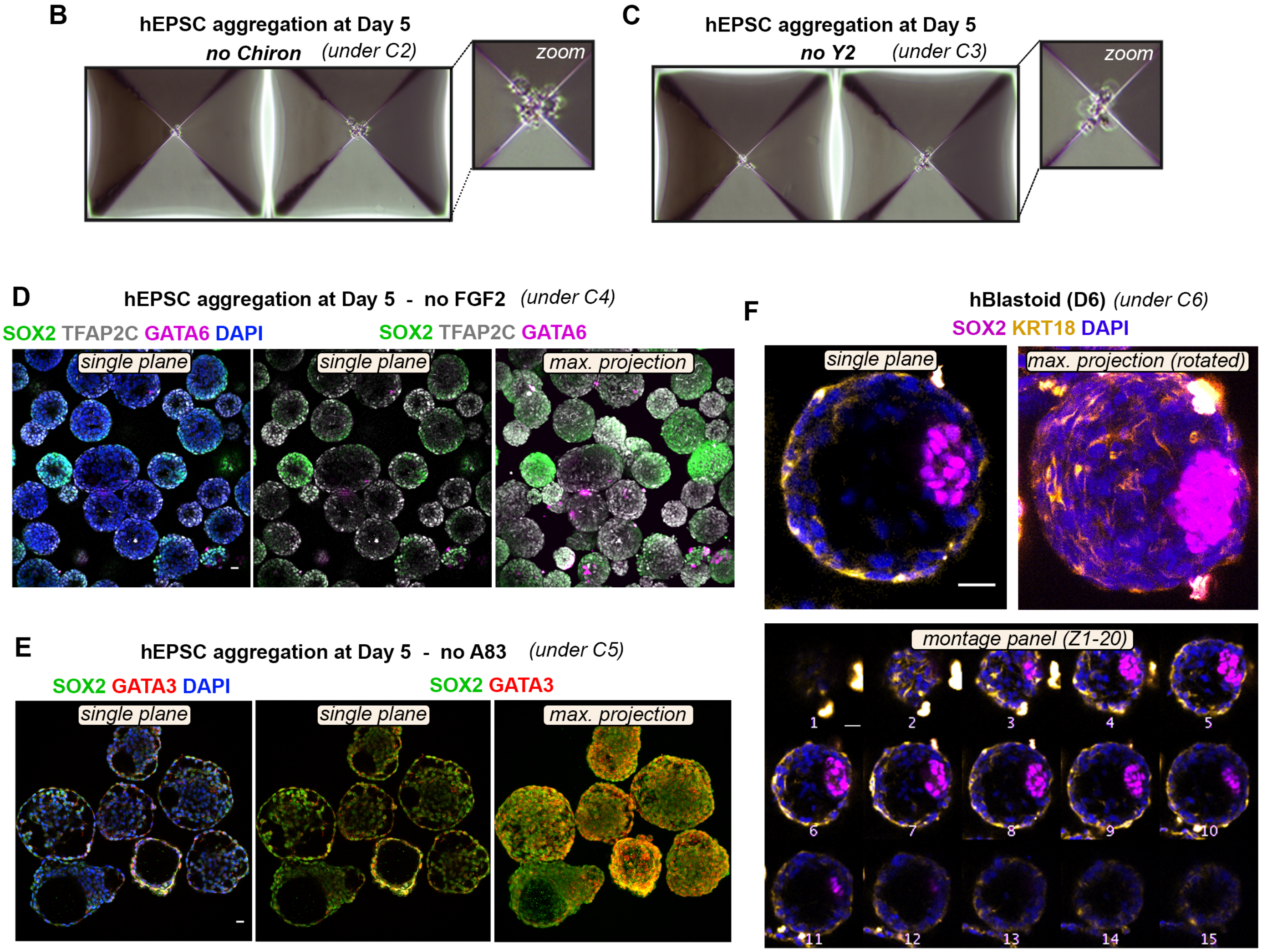

Supplementary Figure 2. Screening of growth factors and inhibitors on hEP-derived structures. A. Table outlining tested media conditions with and without specific growth factors and inhibitors (left), and observed features from each of these conditions (right). **B, C.** Images show failure of cell survival grown for 5 days without CHIR99021 (Chiron, condition 2, C2) or Y-27632 (Y2; condition 3, C3), respectively. **D.** Immunostaining showing expression of TFAP2C (white), and GATA6 (magenta) in structures grown under condition 4 (C4) for 5 days. DAPI shows nuclear staining in blue. Representative of at least 2 independent experiments. **E.** Immunostaining showing expression of GATA3 (red), and SOX2 (green) in structures grown under condition 5 (C5) for 5 days. DAPI shows nuclear staining in blue. Representative of at least 2 independent experiments. **F.** Immunostaining for SOX2 (magenta), and KRT18 (yellow) in representative structure grown under condition 6 (C6) until Day 6. Images are shown in a single plane view (top left), maximum projection (top right), and as a montage panel of Z-stack slice 1-20. All scale bars in the figure indicate 20 um. Representative of at least 2 independent experiments.

Supplementary Figure 3

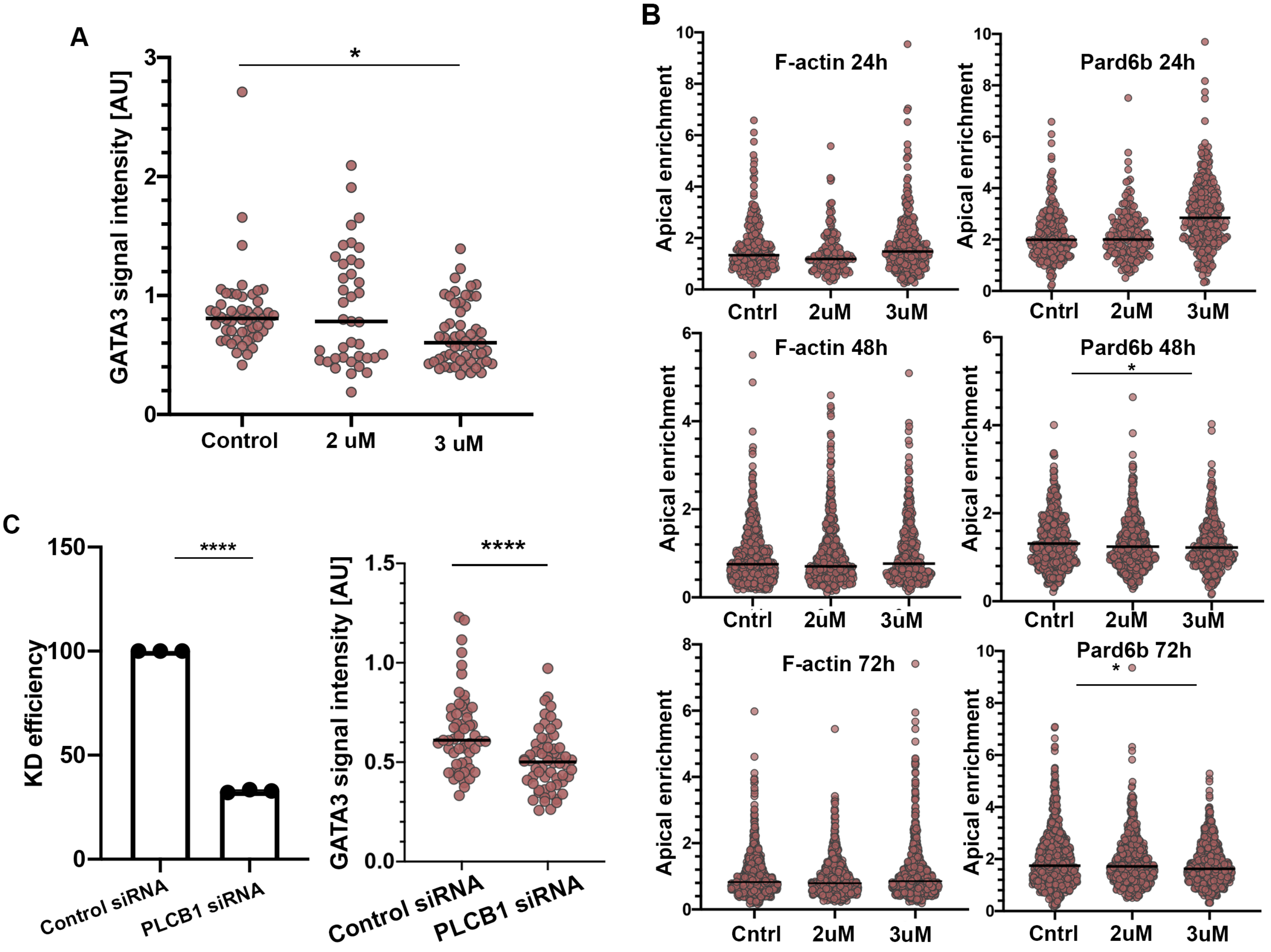

Supplementary Figure 3. PLC-Protein Kinase C (PKC) pathway suppression affects polarity establishment. A. Quantification of nuclear GATA3 signal intensity in control and experimental groups treated with either 2uM or 3uM of selective PKC inhibitor, U73122. All measurements normalized to DAPI. Each dot represents one analysed cell. p=0.0173, ANOVA with a multiple comparisons test. Data is shown as mean S.E.M. n=6 aggregates, 3 experiments. **B.** Apical enrichment quantification of F-ACTIN and PARD6b at 24, 48h and 72h in multicellular structures with or without addition of PLC inhibitor (U73122). Control groups received no inhibitor, while the two experimental groups were treated with either 2uM or 3uM U73122. Each dot represents one analysed cell. p=0.0033 for Pard6b 48h; p=0.0267 for Pard6b 72h, Kruskal-Wallis test with Dunn's multiple comparisons test. Error bars show S.E.M. n= n=300 aggregates, 3 experiments. **C. Left:** Quantification of PLCB1 knock-down efficiency in groups treated with either control siRNA or *PLCB1* siRNA as determined by RT-PCR. Values were normalized against GAPDH. ****p<0.001, Two-sided Student's t-test. Approximately 800 structures were pooled for RT-PCR per group from 3D culture. n = 3 replicates. Error bars show S.E.M. **Right:** Quantification of nuclear GATA3 signal intensity in groups treated with either Control siRNA or *PLCB1* siRNA. All measurements normalized to DAPI. Each dot represents one analysed cell. ****p<0.001, Two-sided Student's t-test. n=6 aggregates, 3 experiments. Error bars show S.E.M.

Supplementary Figure 4

A. Pluripotency markers: Expression in EPSCs, Synthetic, Natural

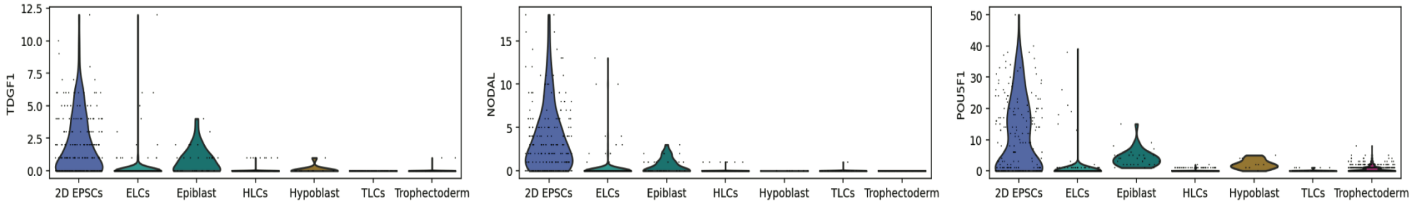

B. Antibodies: Expression in EPSCs, Synthetic, Natural

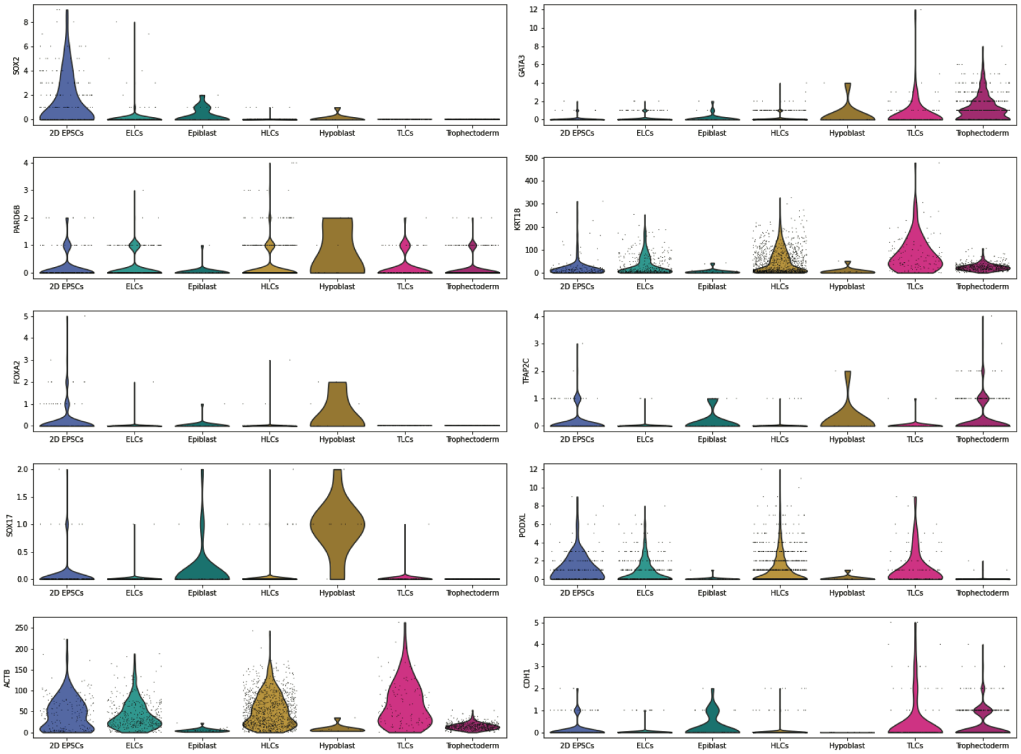

C. Genes from Fig. 5: Expression in EPSCs, Synthetic, Natural

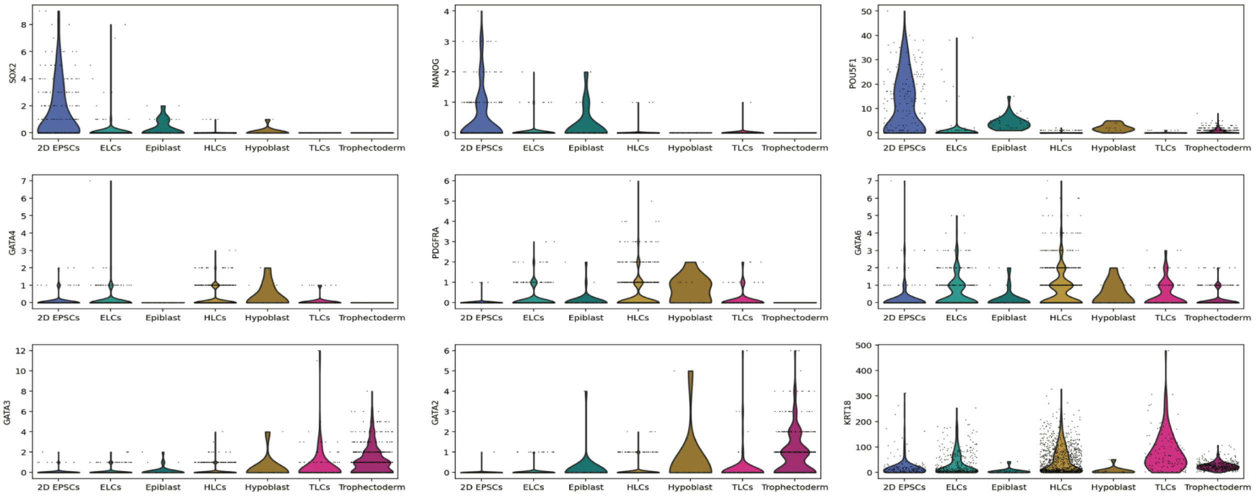

D. EPSC score in 2D hEPSCs vs. Natural embryo

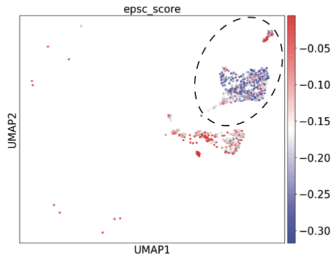

E. EPSC score in 2D hEPSCs, hEP-structures, and Natural embryo

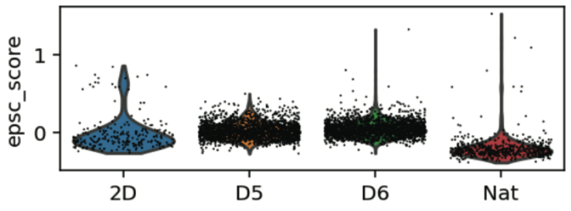

Supplementary Figure 4. Characterization of hEPSCs in 2D culture with scRNA-seq analysis. A. Violin plots showing the expression of key pluripotency markers in 2D hEPSCs, lineages from hEP- derived structures, and lineages from natural embryos. **B.** Violin plots showing the expression of all genes corresponding to antibodies used throughout the main text in 2D hEPSCs, lineages from hEP- structures, and lineages from natural embryos. **C.** Violin plots showing the expression of genes used in Figure 5 in 2D hEPSCs, lineages from hEP- structures, and lineages from natural embryos. **D.** Heatmap of UMAP projection including cells from 2D hEPSCs and natural embryos showing relative "EPSC scores" (See Methods). Cells from natural embryo are surrounded by the circle with a grey dotted line. **E.** Violin plots comparing the "EPSC score" in 2D hEPSCs, D5 hEP-structures, D6 hEP- structures, and natural embryo. For 2D hEPSCs: n = 228 cells, 2 replicates. For natural embryos: n = 542 cells, 6 embryos.

Supplementary Figure 5

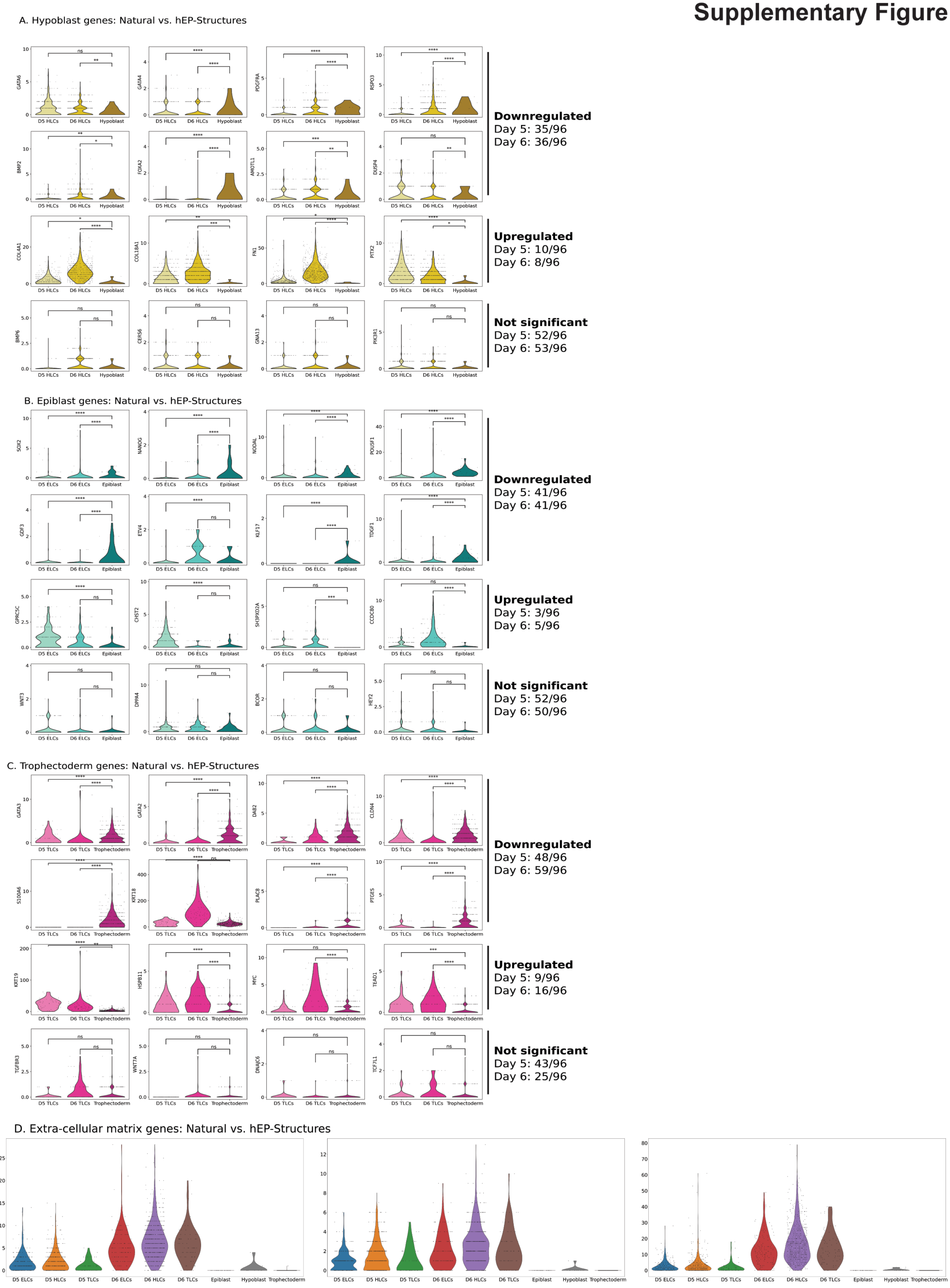

Supplementary Figure 5. Characterization and statistical significance of assigned lineage scores compared to natural embryo. A. Violin plots showing the expression of HYPO-related genes in Day 5 HLCs, Day 6 HLCs, and natural blastocyst HYPO lineage. **B.** Violin plots showing the expression of EPI-related genes in Day 5 ELCs, Day 6 ELCs, and natural blastocyst EPI lineage. **C.** Violin plots showing the expression of TE-related genes in Day 5 ELCs, Day 6 ELCs, and natural blastocyst TE. **D.** Violin plots showing the expression of extra-cellular matrix genes in defined lineages of D5/D6 structures and lineages of natural embryos. Statistical analysis (two-sided ad-hoc Dunn's multiple comparison test applied to an ANOVA) was performed on 96 genes for each lineage, and the fraction of downregulated, upregulated, and not significant genes in comparison to the natural embryo are shown to the right of violin plots. (*$p < 0.05$, **$p < 0.01$, ***$p<0.001$; exact p-values are listed in Supp. Data 2). For Day 5 structures, n = 2013 cell, 3 replicates. For Day 6 structures, n = 2057 cell, 3 replicates. For natural blastocyst, n = 542 cell, 6 embryos.

Supplementary Figure 6.

A.

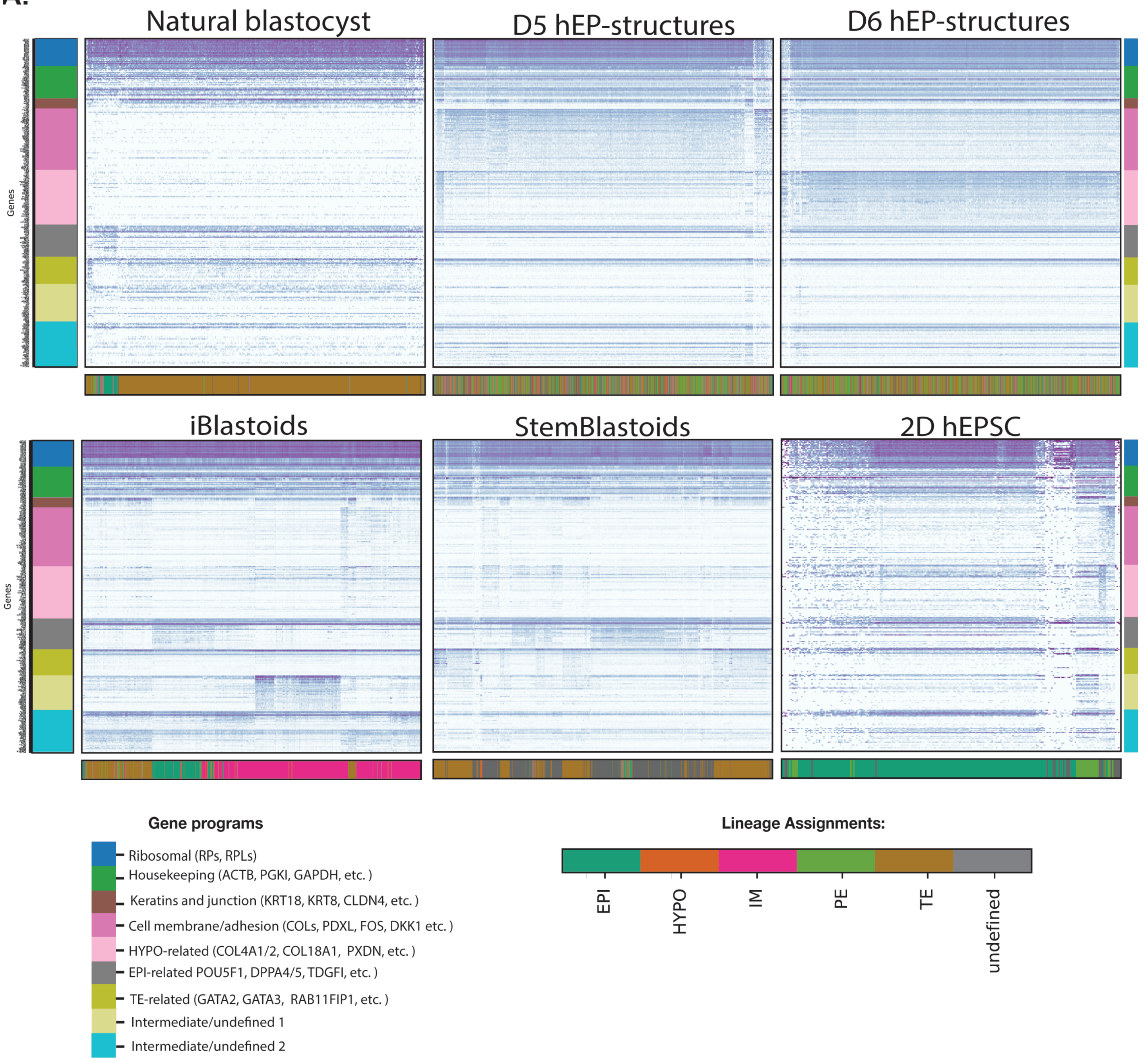

B.

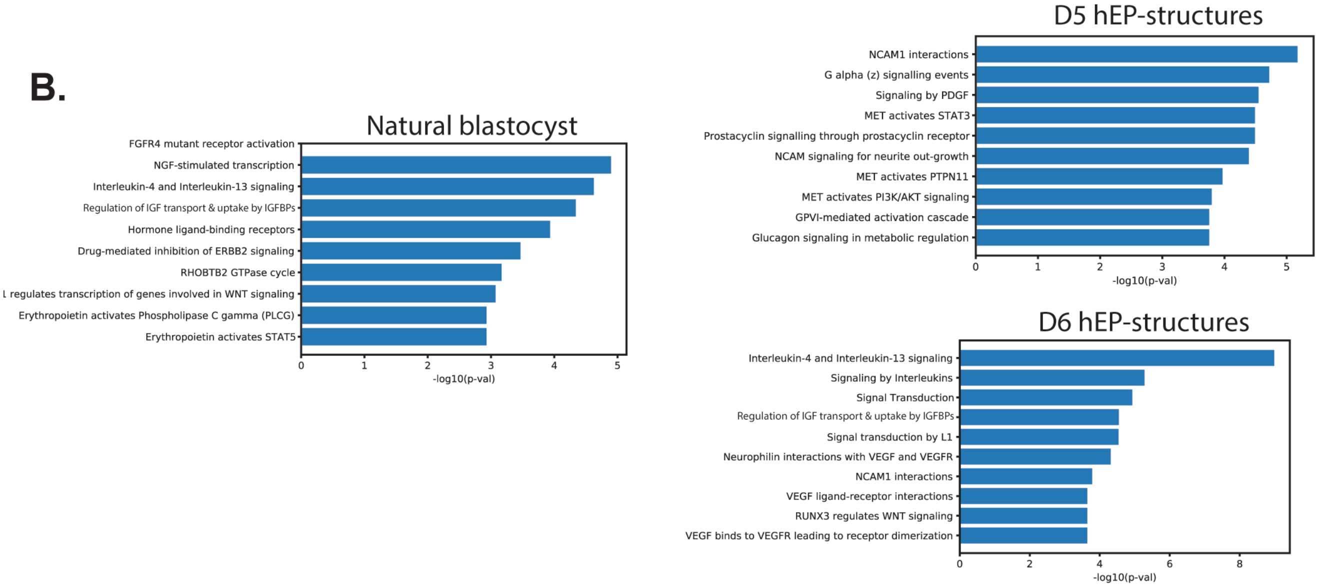

Supplementary Figure 6. Global analysis and comparison of hEP-structures, natural embryo, and previously published blastocyst-like models. A. Heat maps showing global gene expression patterns in Day 5 hEP-structures, Day 6 hEP-structures, natural blastocyst, iBlastoids (Liu et al., 2021), StemBlastoids (Yu et al, 2021), and 2D hEPSCs. An identical list of genes is denoted to the left of each heatmap, with a colour block corresponding to related gene groups. Colour bars below each heatmap denotes lineage assignments, as reported in their original publications. **B.** Gene set enrichment analysis based on the hypergeometric test on Day 5 hEP-structures, Day 6 hEP- structures, and natural embryos was performed using Reactome-signaling gene sets to identify specific signaling pathways that are up-regulated in each.

Supplementary Figure 7

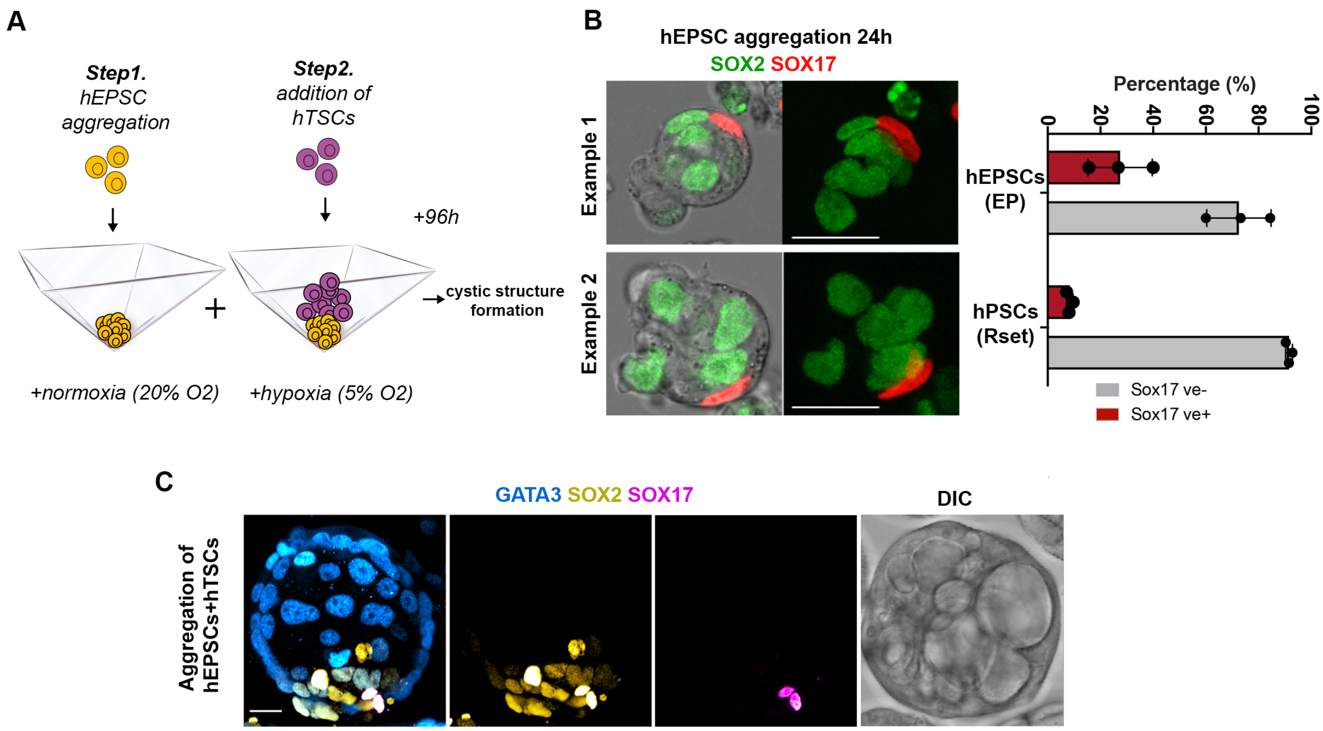

Supplementary Figure 7. Co-assembly of hEPSCs and hTSCs. A. 2-step protocol for generating blastocyst-like structures from EPSCs combined with hTSCs. **B.** Aggregates co-expressing endogenous SOX2 (green) and SOX17 (red) after the first step (24h) of aggregation. SOX2 indicates naive pluripotent EPI-like cells; SOX17 indicates extra-embryonic HYPO-like cell formation. Quantification on the right shows structures scored as positive for hypoblast-like cell in conventional naïve Rset condition and EP condition. n = 3 experiments, 100 aggregates per group. Error bars show S.D. **C.** A representative structure generated from EPSCs combined with hTSCs stained for GATA3, SOX2 and SOX17. DIC image reveals the failure for cavitation. Representative of at least 10 independent experiments. All scale bars in the figure indicate 20 um.

Supplementary Table 1: List of Primary Antibodies

Targeted Protein	Species	Dilution	Catalog No.	Vendor
**PARD6**	Rabbit	1:200	sc-166405	Santa Cruz Biotechnology
**E-Cadherin (Clone 36)**	Mouse	1:200	610182	BD Biosciences
**OCT3/4**	Rabbit	1:200	sc-9081	Santa Cruz Biotechnology
**SOX2**	Rabbit	1:200	D6D9	Cell Signaling Technologies
**GATA3**	Goat	1:200	AF2605	R&D Systems
**PODXL**	Mouse	1:20	MAB1658	R&D Systems
**SOX17**	Goat	1:200	AF1924	R&D Systems
**FOXA2**	Rabbit	1:200	D56D6	Cell Signaling Technologies
**GATA6**	Goat	1:200	AF1700	R&D Systems
**Alexa Fluor® 488 Phalloidin (F-ACTIN)**	N/A	1:500	A12379	Thermo Fisher Scientific
**TFAP2C**	Goat	1:200	AF5059	R&D Systems
**KRT18**	Mouse	1:200	Ab668	Abcam
**Anti-GFP**	Rat	1:1000	04404-84	Nacalai Tesque

Supplementary Table 2: List of Primers for RT-qPCR

Gene	FW Primer	RV Primer
**GATA3**	AAGGCATCCAGACCAGAAACCG	AGCATCGAGCAGGGCTCTAACC
**PLAC8**	GTTTCACCATCTTGGTCAGG	CTGTAATTCCAGCACCTTGG
**CDX2**	CCGAACAGGGACTTGTTTAGAG	CTCTGGCTTGGATGTTACACAG
**KRT8**	ACCCTCAACAACAAGTTTGCCTCC	TCCACTTGGTCTCCAGCATCTTGT
**KRT18**	ACACAGTCTGCTGAGGTTGGAG	TGCTCCATCTGTAGGGCGTAG
**NANOG**	ACACTGGCTGAATCCTTCCTCTCC	CGCTGATTAGGCTCCAACCATACTC
**POU51F**	TCTCGCCCCCTCCAGGT	GCCCCACTCCAACCTGG
**KLF4**	CCCACATGAAGCGACTTCCC	CAGGTCCAGGAGATCGTTGAA
**SOX17**	GAGCCAAGGGCGAGTCCCGTA	CCTTCCACGACTTGCCCAGCAT
**PDGFRA**	CTCCCTGGCTGTTCTGATCG	TGCCAACCCTGTTCCAAAGT
**PLCB1**	GGAAGCGGCAAAAAGAAGCTC	CGTCGTCGTCACTTTCCGT
