# Supplementary material for: Reconstructing aspects of human embryogenesis with pluripotent stem cells
Source: Nat Commun. 2021 Sep 21;12:5550. doi: 10.1038/s41467-021-25853-4 (PMC8455697; doi:10.1038/s41467-021-25853-4)
Supplement: Supplementary file 2 — Reporting Summary [file 41467_2021_25853_MOESM2_ESM.pdf]

nature research

Corresponding author(s): Magdalena Zernicka-Goetz

Last updated by author(s): 25/07/2021

Reporting Summary

Nature Research wishes to improve the reproducibility of the work that we publish. This form provides structure for consistency and transparency in reporting. For further information on Nature Research policies, see our Editorial Policies and the Editorial Policy Checklist.

Statistics

For all statistical analyses, confirm that the following items are present in the figure legend, table legend, main text, or Methods section.

n/a	Confirmed	
☐	☒	The exact sample size (*n*) for each experimental group/condition, given as a discrete number and unit of measurement
☐	☒	A statement on whether measurements were taken from distinct samples or whether the same sample was measured repeatedly
☐	☒	The statistical test(s) used AND whether they are one- or two-sided<br>*Only common tests should be described solely by name; describe more complex techniques in the Methods section.*
☒	☐	A description of all covariates tested
☒	☐	A description of any assumptions or corrections, such as tests of normality and adjustment for multiple comparisons
☐	☒	A full description of the statistical parameters including central tendency (e.g. means) or other basic estimates (e.g. regression coefficient) AND variation (e.g. standard deviation) or associated estimates of uncertainty (e.g. confidence intervals)
☐	☒	For null hypothesis testing, the test statistic (e.g. *F*, *t*, *r*) with confidence intervals, effect sizes, degrees of freedom and *P* value noted<br>*Give P values as exact values whenever suitable.*
☒	☐	For Bayesian analysis, information on the choice of priors and Markov chain Monte Carlo settings
☒	☐	For hierarchical and complex designs, identification of the appropriate level for tests and full reporting of outcomes
☒	☐	Estimates of effect sizes (e.g. Cohen's *d*, Pearson's *r*), indicating how they were calculated

Our web collection on statistics for biologists contains articles on many of the points above.

Software and code

Policy information about availability of computer code

Data collection	Immunofluorescence data was collected using LAS X software (version 3.5.2.18963).
Data analysis	Fiji Image J (version: 2.0.1) open access software, GraphPad Prism (Version 8.4.3 (471))

For manuscripts utilizing custom algorithms or software that are central to the research but not yet described in published literature, software must be made available to editors and reviewers. We strongly encourage code deposition in a community repository (e.g. GitHub). See the Nature Research guidelines for submitting code & software for further information.

Data

Policy information about availability of data

All manuscripts must include a data availability statement. This statement should provide the following information, where applicable:

- Accession codes, unique identifiers, or web links for publicly available datasets
- A list of figures that have associated raw data
- A description of any restrictions on data availability

All relevant data are available from the authors.
The scRNA-seq data for 2D hEPSCs, hEP-structures, and natural human blastocyst generated in this study have been deposited in the GEO database under accession code GSE178326 [GEO, https://www.ncbi.nlm.nih.gov/geo/query/acc.cgi?acc=GSE178326]. Published iBlastoid and StemBlastoid datasets used in this study were obtained from Liu et al. and Yu et al. under accession numbers GSE156596 and GSE150578, respectively
The code generated in this study is provided at https://github.com/vjorgensen/hEP-structures_MZG.
The source data underlying main and and Supplementary Figures are provided as a Source Data file.

Field-specific reporting

Please select the one below that is the best fit for your research. If you are not sure, read the appropriate sections before making your selection.

- [x] Life sciences
- [ ] Behavioural & social sciences
- [ ] Ecological, evolutionary & environmental sciences

For a reference copy of the document with all sections, see nature.com/documents/nr-reporting-summary-flat.pdf

Life sciences study design

All studies must disclose on these points even when the disclosure is negative.

Sample size	No sample-size calculation was performed. Sample size was determined based on previous experimental evidence. For example: Sozen et al, Nature Cell Biology, 2018 and Sozen et al, Developmental Cell, 2019. The experiments were not randomized and the investigators were not blinded to allocation during experiments and outcome assessment.
Data exclusions	Culture experiments: on principle, data were only excluded for failed experiments, reasons for which included suboptimal culture conditions. Following completion of any given aggregation experiment (from day 4 to 6), all cystic structures those clearly displaying a cavity were included in further analyses. Non-cavitated structures were excluded from downstream analyses.
Replication	Each result described in the paper is based on at least two independent biological replicates but very often an experiment is based on more. Figure legends indicate the number of independent experiments performed in each analysis.
Randomization	For experiments were our samples were exposed to chemical inhibitors, samples were randomly allocated to control and experimental groups.
Blinding	Investigators were not blinded as in most cases the morphology could be distinguished based on embryo.

Reporting for specific materials, systems and methods

We require information from authors about some types of materials, experimental systems and methods used in many studies. Here, indicate whether each material, system or method listed is relevant to your study. If you are not sure if a list item applies to your research, read the appropriate section before selecting a response.

Materials & experimental systems

n/a	Involved in the study	
[ ]	[x]	Antibodies
[ ]	[x]	Eukaryotic cell lines
[x]	[ ]	Palaeontology and archaeology
[x]	[ ]	Animals and other organisms
[x]	[ ]	Human research participants
[x]	[ ]	Clinical data
[x]	[ ]	Dual use research of concern

Methods

n/a	Involved in the study	
[x]	[ ]	ChIP-seq
[x]	[ ]	Flow cytometry
[x]	[ ]	MRI-based neuroimaging

Antibodies

Antibodies used

PARD6 Rabbit 1:200 sc-166405 Santa Cruz Biotechnology
E-Cadherin (Clone 36)
Mouse 1:200 610182 BD Biosciences
OCT3/4 Rabbit 1:200 Sc-9081 Santa Cruz Biotechnology
SOX2 Rabbit 1:200 D6D9 Cell Signaling Technologies
GATA3 Goat 1:200 AF2605 R&D Systems
PODXL Mouse clone 222328 1:20 MAB1658 R&D Systems
SOX17 Goat 1:200 AF1924 R&D Systems
FOXA2 Rabbit 1:200 D56D6 Cell Signaling Technologies
GATA6 Goat 1:200 AF1700 R&D Systems
Alexa Fluor® 488 Phalloidin (F-ACTIN) 1:500 A12379 Thermo Fisher Scientific
TFAP2C Goat 1:200 AF5059 R&D Systems
KRT18 Mouse 1:200 Ab668 Abcam
Anti-GFP Rat 1:1000 04404-84 Nacalai Tesque

Validation

The subcellular localization of all the antibodies used in this study has been previously reported iby multiple groups in various human embryo/stem cell studies (shown as immunoflourescence labelling).

Relevant citations:
Cancer Science 100, 2009. DOI: 10.1111/j.1349-7006.2009.01125.x.
Nature Methods 16, 2019. DOI: 10.1038/s41592-018-0253-2.
Development, 138(2011) 3011-3020
Nature Cell Biology 2018 Aug;20(8):979-989. doi: 10.1038/s41556-018-0147-7.
eLife 2018;7:e32839

Eukaryotic cell lines

Policy information about cell lines

Cell line source(s)	The hPSC lines utilized in this study include:<br>-RUES2-GLR (kindly provided by Ali Brivanlou, Rockefeller U., USA)<br>-ESI017 (kindly provided by Juan Michael Elowitz, California Institute of Technology, USA). Commercial Source: ESI BIO https://esibio.com/agex/esi-017-human-embryonic-stem-cell-line-46-xx/<br><br>Human TSCs were kindly provided by Hiroaki Okae and Takahiro Arima<br>(Tohoku University Graduate School of Medicine, Japan).
Authentication	Cells were maintained in conditions to preserve stem cell character and prevent differentiation. Plates were inspected for morphological evidence of differentiation (altered colony morphology in PSC cultures...etc) and plates with differentiated cells were discarded. Furthermore, cell identities were confirmed routinely by immunoflourescence marker expressions.
Mycoplasma contamination	Each of these cell lines was tested negative for mycoplasma contamination, which was monitored and confirmed negative on a bi-monthly basis<br>(MycoScope™ PCR Mycoplasma Detection Kit, Genlantis).
Commonly misidentified lines<br>(See ICLAC register)	The cells we used are not part of this database
