# Supplementary material for: Reconstructing aspects of human embryogenesis with pluripotent stem cells
Source: Nat Commun. 2021 Sep 21;12:5550. doi: 10.1038/s41467-021-25853-4 (PMC8455697; doi:10.1038/s41467-021-25853-4)
Supplement: Supplementary file 3 — Description of Additional Supplementary Files [file 41467_2021_25853_MOESM3_ESM.pdf]

File Name: Supplementary Data 1
Description: List of marker genes used to assign lineages for scRNA-seq analysis.

File Name: Supplementary Data 2
Description: Results of statistical analysis (two-sided ad-hoc Dunn's multiple comparison test applied to an ANOVA) performed on 96 genes for each lineage, and the fraction of downregulated, upregulated, and not significant genes in comparison to the natural embryo. For Day 5 structures, n = 2013 cell, 3 replicates. For Day 6 structures, n = 2057 cell, 3 replicates. For natural blastocyst, n = 542 cell, 6 embryos.
